# Plasticity and immune marker profiling of tumour- and ascites-derived microtumours in serous ovarian cancer: relevance for short-term culture models

**DOI:** 10.3389/fimmu.2026.1901291

**Published:** 2026-10-07

**Authors:** Louisa Hell, Yi Ding, Clara Pellens, Kathrin Eylmann, Maila Rossberg, Carsten Bokemeyer, Jürgen Kupper, Barbara Schmalfeldt, Franziska Brauneck, Jasmin Wellbrock, Tabea Sturmheit, Leticia Oliveira-Ferrer

**Affiliations:** 1 Department of Gynecology, University Medical Center Hamburg-Eppendorf, Hamburg, Germany; 2 2cureX, Copenhagen, Denmark; 3 Department of Oncology, Hematology and Bone Marrow Transplantation with Section Pneumology, Hubertus Wald University Cancer Center, University Medical Center Hamburg-Eppendorf, Hamburg, Germany

**Keywords:** cellular plasticity, immune checkpoint molecules, immune evasion, patient-derived microtumours, serous ovarian cancer, tumour microenvironment

## Abstract

**Introduction:**

Serous ovarian cancer (SOC) is characterized by tumour heterogeneity, cellular plasticity, and immune suppression, contributing to limited therapeutic responses. Understanding tumour -immune dynamics at the cellular level is essential for developing personalized treatment strategies.

**Methods:**

We established a patient-derived microtumour (MT) platform from solid tumour tissue and malignant ascites of 18 treatment-naïve SOC patients. Using multiparametric flow cytometry, we analysed plasticity-associated markers (CD10, CD24, CD44, CD90, CD133), immunoregulatory molecules (PD-L1, PVR, PVRL2, CD47, Calreticulin, CD39, CD73), and antigen presentation molecules (HLA-A/B/C, HLA-DR/DP/DQ) during short-term culture.

**Results:**

Plasticity-associated marker expression was largely conserved across compartments, with substantial interpatient heterogeneity and only modest differences in an exploratory composite plasticity score. Compared with tumour-derived MTs, ascites-derived MTs exhibited significantly higher calreticulin expression and lower expression of the ectonucleotidase CD39. Short-term culture induced CD44 upregulation and CD90 downregulation as well as dynamic modulation of immune markers, such as increased PVR and PVRL2 expression. HLA expression showed substantial interindividual heterogeneity rather than clear compartment-specific differences. Co-expression analyses revealed associations between plasticity-associated and immunoregulatory features, such as CD10/CD24 with PD-L1 and CD47. Finally, exploratory autologous co-cultures demonstrated the feasibility of combining patient-derived MTs with matched immune cells. Two of five samples showed moderate responses to anti-PD-L1 and anti-TIGIT, reflected by increased granzyme B expression and accompanied by compensatory changes in TIGIT and PD-L1 expression.

**Discussion:**

Taken together, these findings indicate an association between tumour cell plasticity and an immune-evasive phenotype in ovarian cancer and support patient-derived MTs as an exploratory short-term platform for capturing patient-specific tumour -immune interactions, while highlighting dynamic immune checkpoint molecule expression during cultivation.

## Introduction

Ovarian cancer (OC) is the eighth most common cancer among women and accounts for approximately 4.8% of cancer-related deaths worldwide (1). Due to the absence of early symptoms, nearly 70% of cases are diagnosed at advanced stages (FIGO III–IV), when the disease has often spread within the peritoneal cavity (2). Consequently, advanced OC is associated with a poor prognosis, with a 5-year survival rate below 30% (3). Treatment typically includes cytoreductive surgery followed by platinum-based chemotherapy (4). Although targeted therapies such as PARP and VEGF inhibitors have expanded treatment options, relapse still occurs in the majority of patients with advanced disease (5). This clinical pattern underscores the need to better understand the biological mechanisms driving therapeutic resistance and recurrence in advanced OC. Tumour heterogeneity and cellular plasticity contribute substantially to therapeutic resistance and disease recurrence. Cancer stem cells (CSCs) represent a subpopulation of tumour cells characterized by enhanced self-renewal capacity and inherent resistance to chemotherapy and are believed to contribute to tumour initiation and relapse (6).

Several CSC-associated markers have been described in OC, including CD10, CD24, CD44, CD90, and CD133, which are linked to tumour progression, metastasis, and therapy resistance. CD10 has been associated with tumour dissemination (7, 8), while CD24 promotes tumourigenicity and immune evasion through interaction with Siglec-10 (9, 10). CD44 contributes to metastasis, CSC maintenance, and chemoresistance (11). CD90 shows context-dependent functions, acting as a CSC marker in several cancers but potentially suppressing stemness in OC (12). CD133 is a well-established CSC marker associated with tumour progression and chemoresistance (13). Notably, such plastic, stemness-associated states are increasingly linked to immune modulation, including altered expression of immune checkpoints and antigen presentation molecules.

Immune checkpoint inhibitors have revolutionized the treatment of many solid tumours, but their efficacy in OC remains limited, showing only modest clinical benefit in unselected OC populations and restricting routine use to specific trial settings or biomarker-defined subgroups. For example, the JAVELIN Ovarian 100 trial did not support avelumab as first-line therapy (14), whereas the DUO-O trial demonstrated improved progression-free survival with durvalumab combined with bevacizumab and olaparib maintenance therapy in advanced OC (15). More recently, the phase III ENGOT-ov65/KEYNOTE-B96 trial demonstrated a survival benefit with pembrolizumab plus paclitaxel in PD-L1-positive platinum-resistant OC, leading to FDA approval of this combination (16). These findings highlight the need for predictive biomarkers to guide immune-based combination therapies.

Beyond the PD-L1/PD-1 axis (17), additional immune regulatory pathways contribute to immune evasion in OC. The PVR-TIGIT pathway is increasingly recognized, with PVR and TIGIT frequently expressed in OC (18). PVRL2 can further suppress immune responses via TIGIT and PVRIG (19). CD47, a “don’t eat me” signal, supports tumour immune escape by inhibiting macrophage-mediated phagocytosis. CD47 inhibits macrophage-mediated phagocytosis, while calreticulin promotes phagocytic clearance (20–23). In addition, the ectonucleotidases CD39 and CD73 generate immunosuppressive adenosine within the tumour microenvironment and have been associated with poor survival in SOC (24). Reduced HLA class I and II expression further impairs antigen presentation and contributes to immune escape (25, 26).

An additional challenge in advanced OC is its pronounced intrapatient heterogeneity. At late stages, primary tumours, metastatic lesions, and malignant ascites coexist, each representing distinct tumour microenvironments (TMEs) that can differentially influence tumour progression and CSC behaviour (27).

In this study, we analysed stemness- and immune-related markers in serous epithelial OC microtumours derived from solid tumour tissue and malignant ascites. By comparing these compartments, we aimed to identify differences reflecting functional heterogeneity among tumour cell subsets and to explore their potential relevance for immune-based therapeutic strategies. In addition, selected markers were used to establish autologous immune co-culture models with patient-derived MTs, enabling functional testing of immunomodulatory approaches.

## Materials and methods

### Patient cohorts

Solid tumour tissue and malignant ascites samples were collected from 20 treatment-naïve patients during primary debulking surgery and/or ascites puncture. Following histopathological evaluation, two patients with endometrial carcinoma were excluded (**Supplementary Figure 1A**). Samples from 18 treatment-naïve patients with SOC were processed for microtumour preparation as outlined in **Supplementary Figure 1B**. The study was conducted in accordance with the Declaration of Helsinki and approved by the Ethics Committee of the Hamburg Medical Association (PV6012-4312-BO-ff). Clinicopathological characteristics, including FIGO stage and TNM classification, are provided in **Supplementary Table 1**. In addition to the 18 patients used for phenotypic microtumour analyses, tumour tissue from five additional patients was included exclusively in autologous co-culture experiments.

### Preparation of MTs derived from solid tumour tissue and malignant ascites

Primary tumour cells were isolated from fresh tumour tissue by mechanical dissociation and rinsing with phosphate-buffered saline (PBS) (Sigma-Aldrich, St. Louis, Missouri, USA) supplemented with 0.1% bovine serum albumin (BSA) (Sigma-Aldrich, St. Louis, Missouri, USA). MTs with a size of 40–100 µm were obtained by sequential filtration using a 100 µm cell strainer placed on top of a 40 µm cell strainer (Greiner Bio-One, Kremsmünster, Austria). MTs retained in the 40 µm strainer were collected and set aside. Tissue fractions larger than 100 µm were enzymatically digested at 37 °C for 30 minutes with 1 mg/ml Collagenase II (Gibco, Thermo Fisher Scientific, Waltham, Massachusetts, USA) in Advanced DMEM/F-12 (Gibco, Thermo Fisher Scientific, Waltham, Massachusetts, USA) supplemented with 10 mM HEPES (Gibco, Thermo Fisher Scientific, Waltham, Massachusetts, USA) and 0.1% BSA (Sigma-Aldrich, St. Louis, Missouri, USA). The digested material was resuspended, filtered as described above, and pooled with previously collected MT fractions.

MT preparations (40–100 µm) were counted and divided into two groups: one was immediately cryopreserved on day 0 (d0), while the other was cultured in MT growth medium as described by Narasimhan et al. (28) on agarose-coated 60 mm Petri dishes (Sarstedt AG & Co. KG, Nümbrecht, Germany) before cryopreservation on day 7 (d7). All samples were subsequently analysed by flow cytometry.

Similarly, MTs of 40–100 µm were isolated from freshly obtained ascites samples of OC patients using 100 µm and 40 µm cell strainers (Greiner Bio-One, Kremsmünster, Austria). The isolated fraction was counted, partially cryopreserved, and partially cultured under identical conditions on agarose-coated 60 mm Petri dishes (Sarstedt AG & Co. KG, Nümbrecht, Germany) for seven days prior to flow cytometric analysis (**Supplementary Figure 1B**). Depending on the quality and amount of tumour tissue or ascites available, between 5,000 and 1,030,000 MTs were obtained per sample (median 85,000).

In total, 51 MT samples from 18 SOC patients were analysed by flow cytometry (see **Supplementary Figure 1A**).

### Multiparameter flow cytometry

Cryopreserved MT samples were thawed, enzymatically dissociated with Accutase at 37 °C for 10 minutes (Sigma-Aldrich) and stained for multiparameter flow cytometry. After Fc blocking with Human TruStain FcX™ Fc Receptor Blocking Solution (BioLegend, San Diego, CA, USA), cells were stained with the viability dye Invitrogen™ Pacific Orange™ Succinimidyl Ester (Thermo Fisher Scientific, Waltham, MA, USA) to exclude dead cells. Surface staining was performed using fluorochrome-conjugated antibodies listed in **Supplementary Table 2A**. Samples were subsequently fixed and permeabilized using the FIX & PERM™ Cell Permeabilization Kit (Thermo Fisher Scientific). Data were acquired on BD FACSymphony A3 using FACS Diva version 8 (BD Biosciences, San Jose, CA, USA).

Data analysis was performed using FlowJo version 10.4 (Treestar, Ashland, OR, USA). Sequential gating excluded debris and dead cells prior to identification of viable CD45^−^EpCAM^+^ MT cells. Fluorescence minus one (FMO) controls were used to define positivity thresholds for HLA-A/B/C and HLA-DR/DP/DQ. Uniform Manifold Approximation and Projection (UMAP) was performed in FlowJo for dimensionality reduction and visualization of multiparametric datasets.

After exclusion of duplicates and samples with <200 events, n = 14 d0 and n = 10 d7 MT specimens from solid tumour tissue and n = 8 d0 and n = 7 d7 MT specimens from malignant ascites were included in the final analyses (**Supplementary Figure 1A**).

Representative gating strategies and individual flow-cytometry plots for all analysed cell populations and markers are provided in **Supplementary Figure 2**. Corresponding fluorescence-minus-one (FMO) controls are shown where these were used to define marker-positive populations.

### Autologous co-culture for functional immune testing in SOC

An autologous co-culture model was established to explore the technical feasibility of assessing T-cell-associated responses to PD-1 and TIGIT blockade. To ensure sufficient immune cell content, the smallest filtration fraction (<40 µm) was first analysed by flow cytometry in 14 SOC samples to determine the frequency of viable EpCAM^−^CD45^+^ immune cells. Five patient-derived samples with sufficient material were included in the autologous co-culture experiments. Prior to co-culture, baseline expression of PD-1 and TIGIT was assessed on viable CD45^+^CD3^+^CD8^+^ T cells from these samples.

Following filtration, single cells from the <40 µm fraction were cultured for seven days in T-cell medium (TCM) consisting of RPMI-1640 (Thermo Fisher Scientific) supplemented with 10% human serum (Sigma-Aldrich, St. Louis, MO, USA), 1% penicillin/streptomycin (Thermo Fisher Scientific), and 150 U/ml interleukin-2 (IL-2) (PeproTech, Cranbury, NJ, USA) at a concentration of 1.5 × 10^6^ cells/ml in 96-well plates (Sarstedt AG & Co. KG). Co-stimulation was achieved using plate-bound anti-CD28 (Thermo Fisher Scientific) prepared as described by Dijkstra et al. (29). These culture conditions were selected to support T-cell survival and activation during the seven-day pre-culture period.

In parallel, MTs (40–100 µm fraction) were cultured in MT growth medium for seven days. The parallel seven-day culture period was used to synchronise the immune-cell-containing fraction with the autologous MT culture before co-incubation.

After seven days, the <40 µm cell fraction was harvested and allocated to three treatment groups: (1) control (TCM + IL-2), (2) pembrolizumab (20 µg/ml; University Medical Center Hamburg-Eppendorf pharmacy), (3) anti-TIGIT antibody clone A15153G (50 µg/ml; BioLegend). All three conditions were assessed in five patient-derived samples. Owing to limited sample material, a fourth condition combining pembrolizumab and anti-TIGIT was assessed in two of the five samples. Cells were cultured at 1.5 × 10^6^ cells/ml in anti-CD28-coated 96-well plates and co-cultured with autologous d7 MTs from the 40–100 µm fraction at a concentration of 1 MT/µl.

After 24 hours, intracellular and surface staining was performed as described above to assess cell composition (EpCAM, CD45, CD4, CD8), viability (Live/Dead staining; Invitrogen™ Pacific Orange™ Succinimidyl Ester, Thermo Fisher Scientific), and T-cell activation (IFN-γ and granzyme B) (**Supplementary Table 2B**). Samples were analysed by flow cytometry using a BD LSRFortessa™ (BD Biosciences, San Jose, CA, USA).

### Statistical analyses

Unless otherwise specified, data are presented as mean ± standard deviation. Data distributions were assessed for normality. For comparisons between two groups, we distinguished between paired and unpaired datasets according to the experimental design and sample availability. Where matched samples from the same patient were available, paired analyses were performed using either a paired t-test for approximately normally distributed paired differences or a Wilcoxon matched-pairs signed-rank test for non-normally distributed data. For independent samples, we used either Welch’s t-test for approximately normally distributed data or the Mann–Whitney test for non-normally distributed data.

Where multiple marker-wise comparisons were performed within the same analysis, P values were adjusted for multiple testing using the Holm–Šídák method. For analyses involving more than two groups, we applied the appropriate global parametric or non-parametric test, followed by corrected *post-hoc* comparisons where applicable.

Differences among the three treatment conditions assessed in all five matched samples (control, anti-PD-1, and anti-TIGIT) were analysed using a Friedman test followed by Dunn’s multiple-comparisons test. The combination treatment, available for two samples only, was presented descriptively and was not included in the statistical analysis.

A p-value < 0.05 was considered statistically significant.

Plasticity-associated surface marker expression (CD10, CD24, CD90, CD133) was quantified as the percentage of positive cells within d0 CD45^−^EpCAM^+^ MT cells (n = 22; 14 tumour-derived, 8 ascites-derived). Expression values were divided into quartiles across all samples, assigning ordinal scores from 1 (lowest quartile) to 4 (highest quartile). Scores were summed to generate an exploratory composite plasticity score (theoretical range 4–16), with higher scores indicating a relative enrichment of stemness-associated features within the cohort. In the present cohort, observed scores ranged from 7 to 14. Samples were classified into low (7–10) and high (11–14) plasticity groups (**Supplementary Figure 3A**). Pairwise associations between plasticity-marker expression levels were assessed separately in d0 tumour- and ascites-derived MTs using two-sided Spearman correlation tests. P-values were adjusted across the ten marker-pair comparisons within each compartment using the Holm method. HLA-A/B/C and HLA-DR/DP/DQ expression were quantified as MFI in d0 CD45-EpCAM+ MT cells. Cohort-specific median MFIs were calculated separately for both markers, and samples were dichotomized into low (≤ median) and high (>median) expression groups (**Supplementary Figure 3B**).

For clinicopathological correlations, a patient-level plasticity status was assigned based on the composite score. Patients with discordant classifications between tumour- and ascites-derived MTs were excluded. Associations between categorical variables, including patient-level plasticity status and clinicopathological parameters as well as HLA expression category and sample origin, were assessed using Fisher’s exact tests.

## Results

### Plasticity marker profiles of MTs derived from solid tumour tissue and malignant ascites in SOC

Plasticity-associated markers CD10, CD24, CD44, CD90, and CD133 were assessed on viable CD45^−^EpCAM^+^ tumour cells in MTs derived from solid tumour tissue and malignant ascites (**Figure 1A**). CD24 was the most frequently expressed marker in both compartments, with a mean percentage of 75.3% of CD45^−^EpCAM^+^ cells in tumour-derived MTs and 81.7% of ascites-derived MTs presenting this surface marker. CD10 expression was detected on 39.6% of cells from tumour-derived MTs and on 40.4% of cells from ascites-derived MTs. CD44 expression was generally low in both compartments, with 1.1% and 4.0%, respectively. CD133 was present on 18.3% of MT cells from solid tumour tissue and 14.1% of MT cells from malignant ascites.

**Figure 1:**
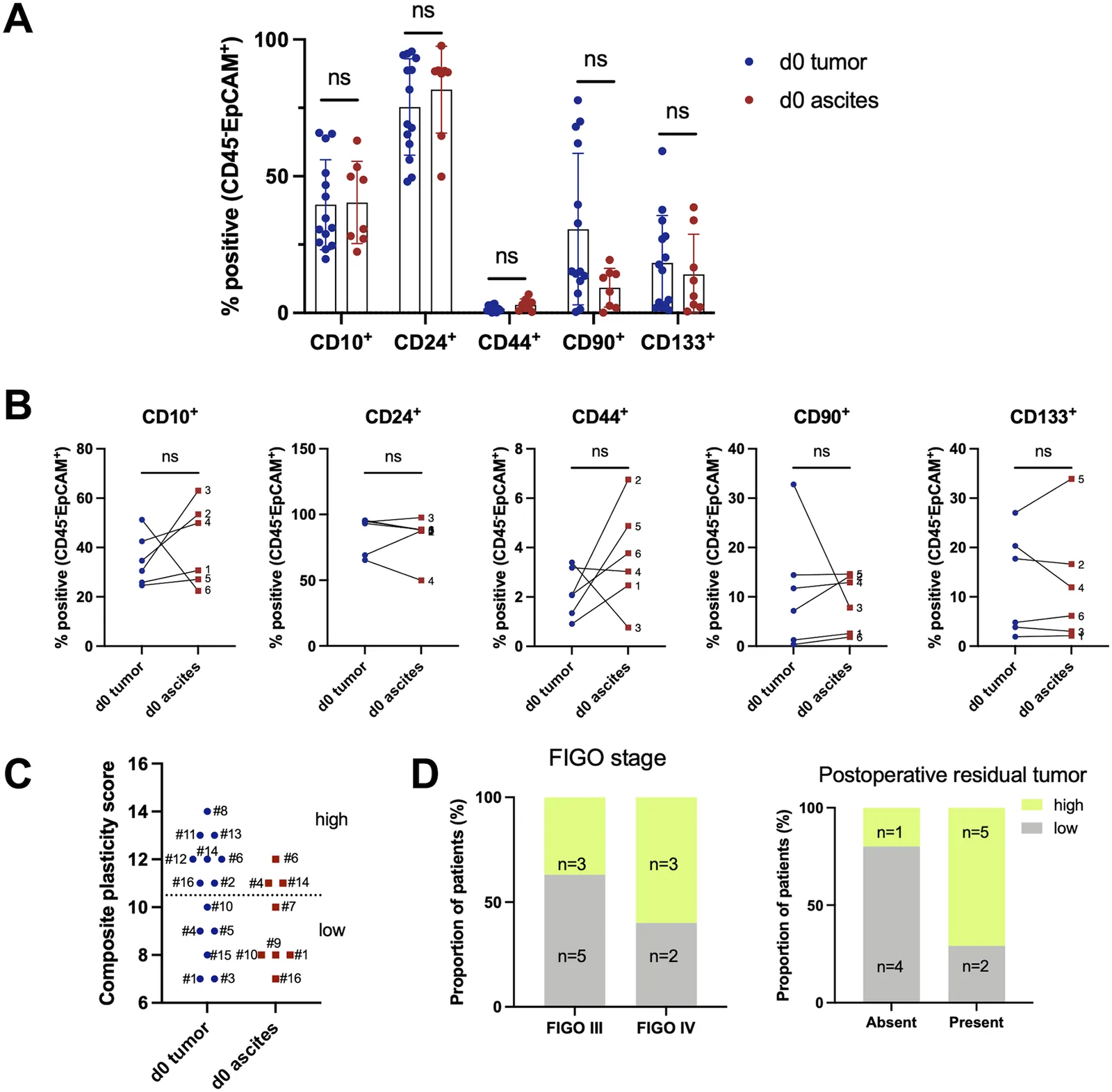
Plasticity marker expression in tumour- and ascites-derived SOC MTs at baseline (d0). **(A)** Percentages of CD10^+^, CD24^+^, CD44^+^, CD90^+^, and CD133^+^ cells within viable CD45^−^EpCAM^+^ MT cells derived from solid tumour tissue and malignant ascites at d0. Blue circles and red squares represent individual tumour- and ascites-derived samples, respectively; bars show mean ± SD. **(B)** Paired comparisons of the percentages of marker-positive cells in matched tumour- and ascites-derived MTs from the same patients (n = 6 pairs). Lines connect matched samples. **(C)** Composite plasticity scores of individual tumour- and ascites-derived d0 MT samples. The dotted horizontal line separates low (7–10) from high (11–14) plasticity scores. **(D)** Distribution of patient-level plasticity status according to FIGO stage (III versus IV) and postoperative residual tumour status (absent versus present). Numbers within the bars indicate patient counts in each category. Patients with discordant plasticity classifications between tumour- and ascites-derived MTs were excluded from the respective patient-level analyses.

No statistically significant differences were observed in the expression of CD10, CD24, CD44, CD133, or CD90 between MT cells derived from solid tumour tissue and malignant ascites. However, CD90 expression showed a trend toward higher and more variable expression in MT cells from solid tumour tissue (0.4–77.8%; mean, 30.6%) than in those from malignant ascites (0.2–19.4%; mean, 9.2%), this difference was not statistically significant. The difference was also not observed consistently in matched samples, indicating substantial interpatient heterogeneity.

To assess relationships between the investigated plasticity-associated markers, pairwise correlations of CD10, CD24, CD44, CD90, and CD133 expression were analysed separately in d0 tumour- and ascites-derived MTs. No pairwise correlation remained statistically significant after correction for multiple testing in tumour-derived MTs. In ascites-derived MTs, CD90 and CD133 showed a strong positive correlation (Spearman’s ρ = 0.93, Holm-adjusted p = 0.0086), whereas the remaining marker pairs were not significantly correlated after adjustment (**Supplementary Figure 4A**). Representative NxN flow-cytometry plots of viable CD45^−^EpCAM^+^ cells illustrated heterogeneous and partially overlapping pairwise marker-expression patterns in tumour- and ascites-derived MTs (**Supplementary Figures 4B, C**).

Matched-sample analysis revealed no significant differences in the proportions of cells positive for any of the investigated plasticity-associated markers between tumour- and ascites-derived MTs, although considerable interpatient variability was observed (**Figure 1B**).

An exploratory composite plasticity score incorporating the percentages of cells positive for CD10, CD24, CD90, and CD133 was calculated as described in the Methods, and samples were classified into low- and high-plasticity groups (**Supplementary Figure 3A**). This composite score was intended to capture the overall plasticity phenotype, as individual markers alone may only partially reflect this complex and dynamic cellular state.

MTs derived from solid tumour tissue showed slightly higher composite plasticity scores compared to those derived from malignant ascites; however, this difference was not statistically significant (**Figure 1C**). Similarly, although higher plasticity scores were more frequently observed in patients with FIGO stage IV and residual tumour after surgery, and lower scores were associated with patients achieving macroscopic complete resection, these associations did not reach statistical significance (**Figure 1D**).

Taken together, we observed no significant differences in plasticity-associated features between compartments, but rather substantial interpatient variability.

### Dynamics of plasticity marker expression in SOC MTs during *in vitro* culture

Cell viability tended to decrease from d0 to d7 in both tumour-derived (n = 7 pairs) and ascites-derived (n = 5 pairs) MT samples. Among viable single cells, the relative proportion of CD45^+^EpCAM^−^ immune cells generally decreased, whereas the proportion of CD45^−^EpCAM^+^ tumour cells increased. EpCAM MFI within viable CD45^−^EpCAM^+^ cells remained broadly unchanged, indicating no systematic change in EpCAM expression intensity within the analysed epithelial tumour-cell population. However, none of the observed changes in viability or relative cellular composition reached statistical significance after correction for multiple testing (**Supplementary Figure 5)**. The observed enrichment of EpCAM^+^ cells is consistent with the culture conditions used in this study, which were specifically established to support the survival and expansion of tumour cells rather than the maintenance of immune cells. Representative microscopy images acquired on d3 and d5 illustrated the persistence of compact tumour- and ascites-derived MT aggregates during short-term suspension culture (**Figure 2A**).

**Figure 2:**
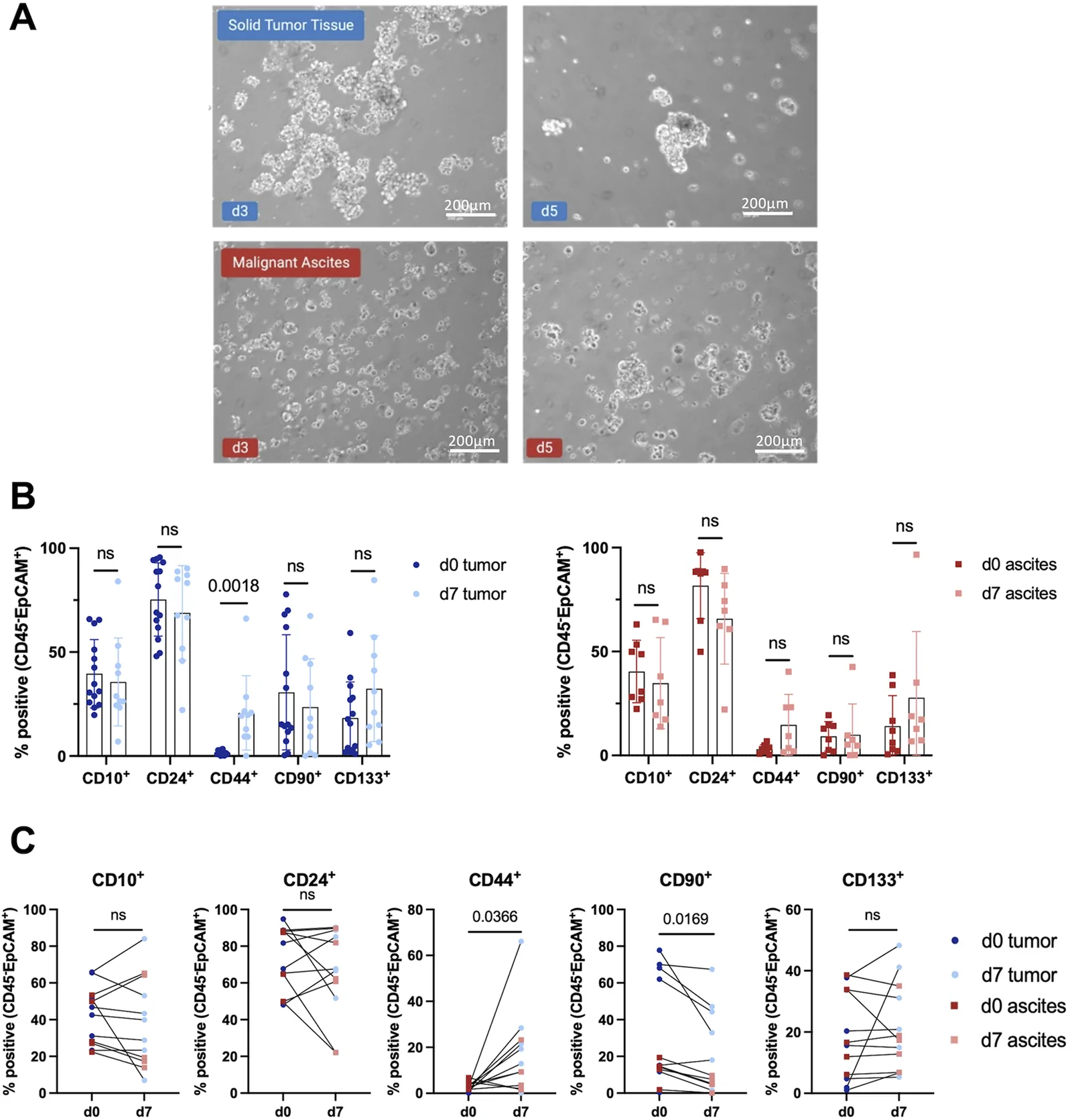
Dynamic changes in SOC MT plasticity marker expression during short-term *in vitro* culture. **(A)** Representative bright-field microscopy images of MTs derived from solid tumour tissue (top) and malignant ascites (bottom) on day 3 (d3) and day 5 (d5) of culture. Day 5 represents the latest time point for which consistent microscopic documentation was available before flow-cytometric analysis on d7. Scale bars represent 200 µm. **(B)** Percentages of CD10^+^, CD24^+^, CD44^+^, CD90^+^, and CD133^+^ cells within viable CD45^−^EpCAM^+^ MT cells at baseline (d0) and after seven days of culture (d7). Tumour-derived MTs are shown on the left (d0, n = 14; d7, n = 10), and ascites-derived MTs on the right (d0, n = 8; d7, n = 7). Bars show mean ± SD, and symbols represent individual samples. **(C)** Paired longitudinal analysis of plasticity-marker expression in matched d0–d7 samples across both compartments (n = 12 pairs: seven tumour-derived and five ascites-derived). Lines connect matched d0 and d7 samples.

In the overall, unpaired analysis, the proportion of CD44^+^ cells was significantly higher in tumour-derived MTs at d7 than at d0 (p = 0.0018). No significant d0–d7 differences were observed for CD10, CD24, CD90, or CD133 in tumour-derived MTs. In ascites-derived MTs, none of the investigated plasticity-associated markers differed significantly between d0 and d7 (**Figure 2B**).

Analysis of the available matched d0–d7 samples across both compartments (n = 12 pairs: seven tumour-derived and five ascites-derived) demonstrated a significant increase in the proportion of CD44^+^ cells (p = 0.0366) and a significant decrease in the proportion of CD90^+^ cells (p = 0.0169). CD10, CD24, and CD133 did not change significantly over time (**Figure 2C**).

In summary, we observed significant changes in the frequency of CD44^+^ and CD90^+^ cells within the EpCAM^+^CD45^−^ population upon cultivation, with an increase in CD44^+^ and a decrease in CD90^+^ cells.

### Analysis of immune marker expression in MTs derived from solid tumour tissue and malignant ascites

Immune marker expression was assessed by measuring MFIs on CD45^−^EpCAM^+^ MTs derived from both solid tumour tissue and malignant ascites (**Figure 3A**). Among the investigated markers, calreticulin and CD39 differed significantly between compartments, with higher calreticulin and lower CD39 expression in ascites-derived compared with tumour-derived MTs (p = 0.0144 and p = 0.0229, respectively). PD-L1, PVR, PVRL2, CD47, and CD73 expression did not differ significantly between tumour- and ascites-derived MTs. Considerable interpatient variability was observed, particularly for CD47, CD39, and CD73.

**Figure 3:**
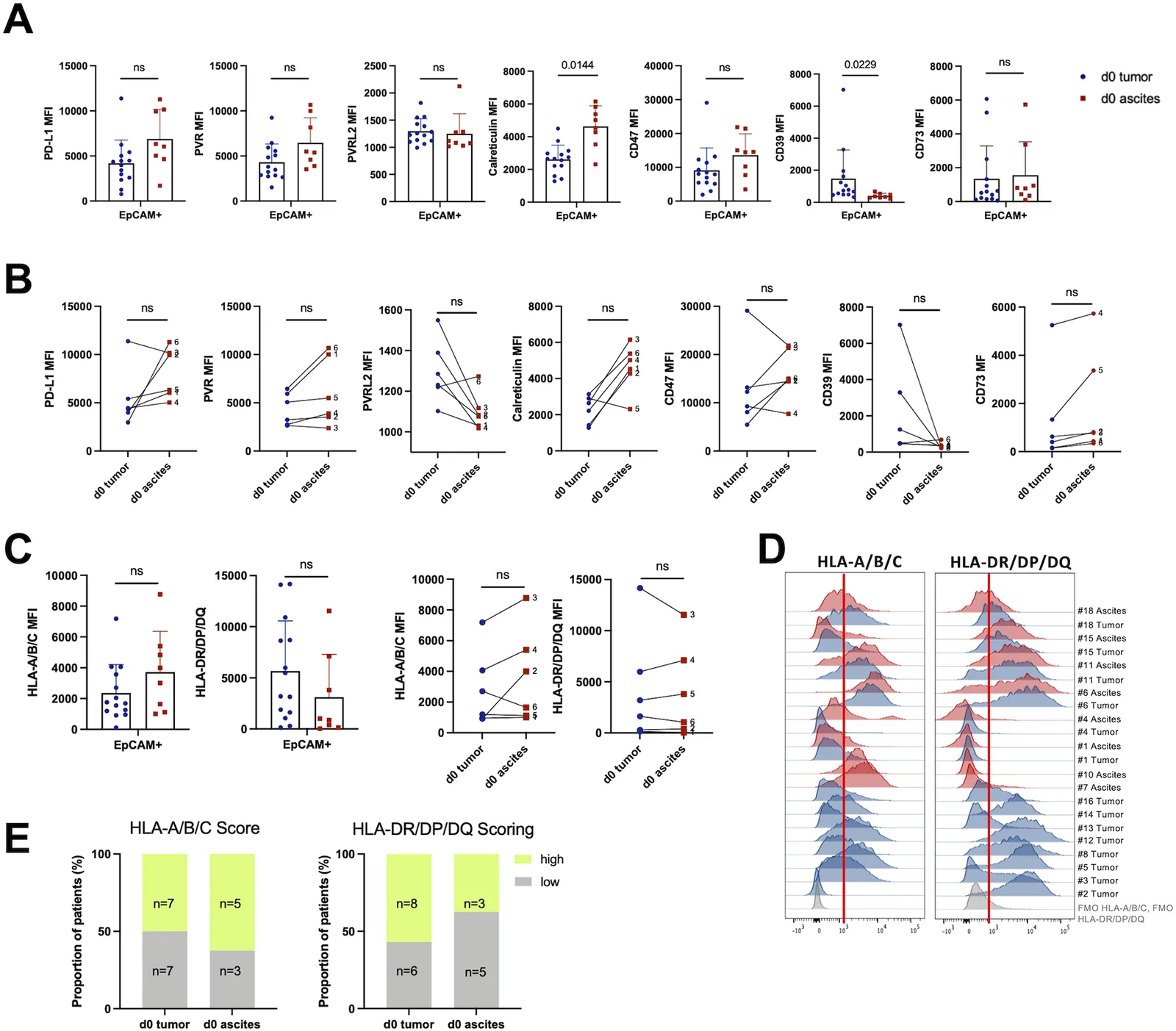
Differential expression of immune-related markers in tumour- vs. ascites-derived SOC MTs at baseline (d0). **(A)** Mean fluorescence intensity (MFI) of PD-L1, PVR, PVRL2, calreticulin, CD47, CD39, and CD73 in viable CD45^−^EpCAM^+^ cells from tumour-derived (blue; n = 14) and ascites-derived (red; n = 8) MTs at d0. Bars show mean ± SD, and symbols represent individual samples. **(B)** Paired comparisons of marker MFI in matched tumour- and ascites-derived MT samples from the same patients (n = 6 pairs). Lines connect matched samples. **(C)** HLA-A/B/C and HLA-DR/DP/DQ MFI in viable CD45^−^EpCAM^+^ cells. Left panels show comparisons of all available tumour-derived (blue; n = 14) and ascites-derived (red; n = 8) samples, with bars indicating mean ± SD and symbols representing individual samples. Right panels show paired comparisons of matched tumour- and ascites-derived samples (n = 6 pairs), with lines connecting samples from the same patient. **(D)** Normalized fluorescence-intensity histograms showing HLA-A/B/C and HLA-DR/DP/DQ expression in individual viable CD45^−^EpCAM^+^ tumour-derived (blue) and ascites-derived (red) MT samples. Grey histograms represent the corresponding fluorescence-minus-one (FMO) controls. Vertical red lines indicate the cohort-specific median MFI thresholds used to classify samples as having low or high HLA expression. **(E)** Proportions of tumour- and ascites-derived MT samples classified as having low or high HLA-A/B/C and HLA-DR/DP/DQ expression based on the cohort-specific median MFI thresholds described in **Supplementary Figure 3B**. Numbers within bars indicate sample counts.

Matched analyses of CD45^−^EpCAM^+^ MT cells from tumour tissue and malignant ascites likewise revealed no statistically significant differences in any of the investigated immunoregulatory markers (**Figure 3B**). Although individual patients showed compartment-specific differences, these patterns were heterogeneous and not consistent across the matched cohort.

In (matched) CD45^−^EpCAM^+^ samples, the MFI of both HLA-A/B/C and HLA-DR/DP/DQ did not differ significantly between MTs derived from tumour tissue versus those from malignant ascites (**Figure 3C**). Furthermore, histogram representations reveal that variations in HLA marker expression are more pronounced between different individuals than between compartments within the same individual (**Figure 3D**).

To further describe HLA expression patterns, samples were classified as having low or high HLA-A/B/C and HLA-DR/DP/DQ expression using cohort-specific median MFI thresholds (**Supplementary Figure 3B**). Tumour-derived MTs were evenly distributed between the low and high HLA-A/B/C categories, whereas ascites-derived MTs showed a slight descriptive enrichment in the high-expression category. For HLA-DR/DP/DQ, high expression was more frequent among tumour-derived MTs, whereas ascites-derived MTs were more frequently classified in the low-expression category. However, no statistically significant associations were observed between sample origin and either HLA-A/B/C or HLA-DR/DP/DQ expression status (**Figure 3E**).

Overall, immunoregulatory marker expression showed substantial interpatient heterogeneity, with higher calreticulin and lower CD39 expression in ascites-derived MTs, indicating compartment-specific differences.

### Dynamics of immune marker expression in MTs during *in vitro* culture

Across all available tumour- and ascites-derived MT samples, PVR and PVRL2 expression increased significantly during short-term culture, with higher MFI values at d7 than at d0 (PVR, p = 0.0001; PVRL2, p = 0.0081). PD-L1, CD47, calreticulin, CD39, and CD73 expression did not differ significantly between d0 and d7 (**Figure 4A**).

**Figure 4:**
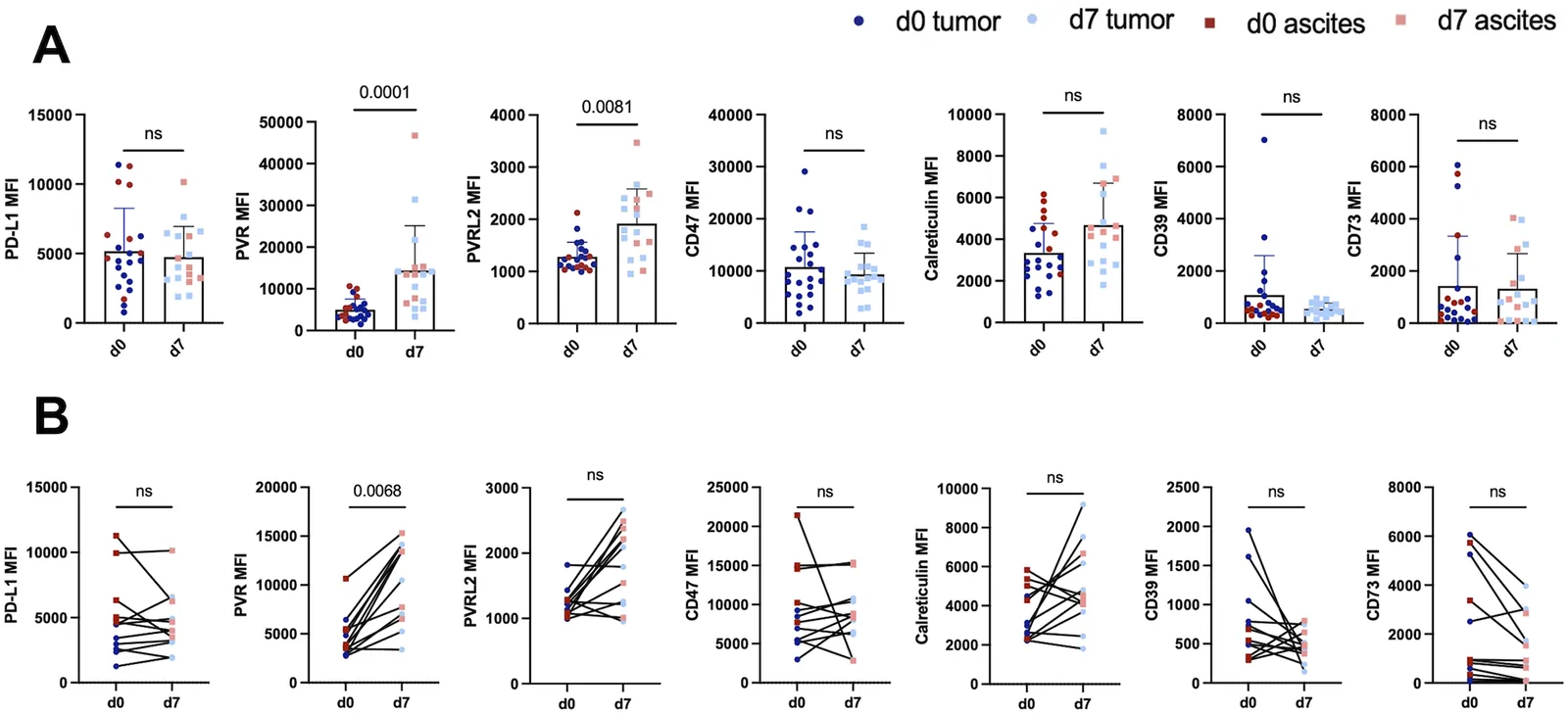
Temporal changes in immune marker expression on SOC MTs during short-term *in vitro* culture. **(A)** Mean fluorescence intensity (MFI) of PD-L1, PVR, PVRL2, calreticulin, CD47, CD39, and CD73 in viable CD45^−^EpCAM^+^ cells at baseline (d0; n = 22) and after seven days of *in vitro* culture (d7; n = 17) across all available tumour- and ascites-derived MT samples. Bars show mean ± SD, and symbols represent individual samples. Dark blue and light blue symbols indicate d0 and d7 tumour-derived samples, respectively; dark red and light red symbols indicate d0 and d7 ascites-derived samples, respectively. **(B)** Paired longitudinal analysis of immunoregulatory marker expression in matched d0–d7 MT samples across both compartments (n = 12 pairs: seven tumour-derived and five ascites-derived). Lines connect matched samples from the same source and patient.

In the longitudinal analysis across matched tumour- and ascites-derived MT samples, the increase in PVR expression from d0 to d7 remained statistically significant (p = 0.0068). Although several samples showed increased PVRL2 expression at d7, this difference was not statistically significant after correction for multiple testing. No significant d0–d7 differences were observed for PD-L1, CD47, calreticulin, CD39, or CD73 (**Figure 4B**). As described above, these longitudinal MFI measurements reflect marker expression within the viable CD45^−^EpCAM^+^ population present at each time point and do not provide information on absolute MT or tumour-cell numbers.

Overall, short-term culture induced significant upregulation of PVR and PVRL2, although only the increase in PVR remained significant in the matched longitudinal analysis.

### Co-expression of plasticity markers and immune markers on MTs derived from solid tumour tissue and malignant ascites

Exploratory UMAP analyses suggested partially overlapping spatial distributions of CD10, CD24, PD-L1, CD47, and calreticulin within viable CD45^−^EpCAM^+^ cells from d0 tumour- and ascites-derived MTs (**Supplementary Figures 6A, B**). Representative bivariate flow-cytometry plots further illustrated the presence of CD10^+^CD24^+^ cells in both compartments (**Figure 5A**). Representative fluorescence-intensity histograms showed shifts in PD-L1, CD47, and calreticulin expression between CD10^−^CD24^−^ and CD10^+^CD24^+^ subpopulations (**Figure 5B**). Within-sample paired analyses demonstrated significantly higher PD-L1, CD47, and calreticulin MFI in CD10^+^CD24^+^ than in CD10^−^CD24^−^ cells in both tumour-derived MTs (PD-L1, p = 0.0008; CD47, p = 0.0004; calreticulin, p = 0.0001) and ascites-derived MTs (PD-L1, p = 0.0011; CD47, p = 0.0011; calreticulin, p = 0.0006) (**Figure 5C**).

**Figure 5:**
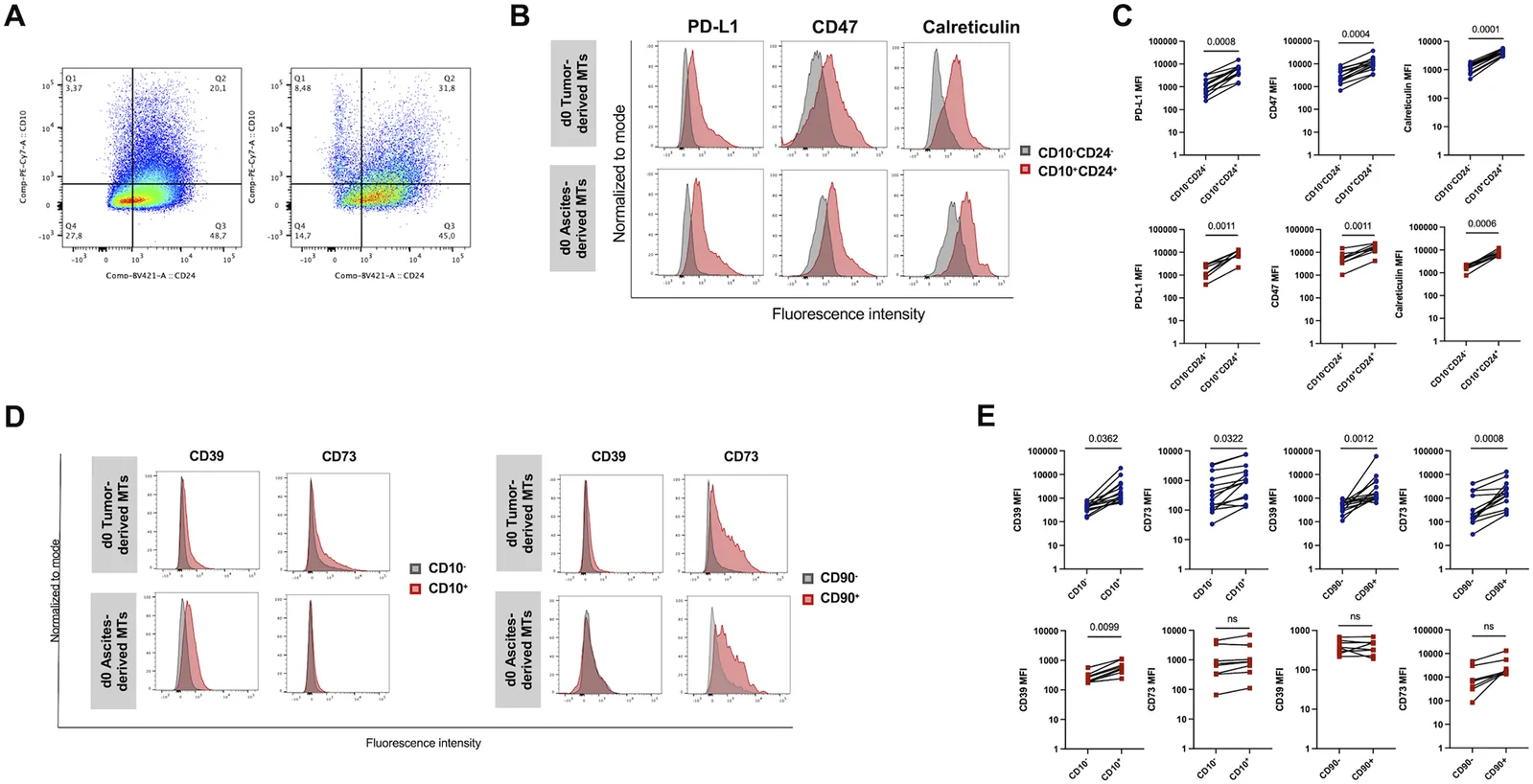
Co-expression and enrichment of immune- and plasticity-associated markers in SOC MTs at baseline (d0). **(A)** Representative flow-cytometry plots showing CD10 and CD24 expression in viable CD45^−^EpCAM^+^ cells from d0 tumour- and ascites-derived MTs. Numbers indicate the percentages of cells within the respective quadrants. **(B)** Representative normalized fluorescence-intensity histograms showing PD-L1, CD47, and calreticulin expression in CD10^−^CD24^−^ (grey) and CD10^+^CD24^+^ (red) viable CD45^−^EpCAM^+^ cells from d0 tumour- and ascites-derived MTs. **(C)** Paired comparisons of PD-L1, CD47, and calreticulin MFI between CD10^−^CD24^−^ and CD10^+^CD24^+^ cells in tumour-derived (blue; n = 14) and ascites-derived (red; n = 8) MTs. Lines connect the two subpopulations from the same sample. **(D)** Representative normalized fluorescence-intensity histograms showing CD39 and CD73 expression in CD10^−^ versus CD10^+^ cells and CD90^−^ versus CD90^+^ cells within viable CD45^−^EpCAM^+^ tumour- and ascites-derived MT cells. Grey and red histograms represent the respective marker-negative and marker-positive subpopulations. **(E)** Paired comparisons of CD39 and CD73 MFI between CD10^−^ and CD10^+^ cells and between CD90^−^ and CD90^+^ cells in tumour-derived (blue; n = 14) and ascites-derived (red; n = 8) MTs. Lines connect the respective subpopulations from the same sample.

Exploratory UMAP analyses additionally suggested partially overlapping distributions of CD10, CD90, CD39, and CD73 within tumour- and ascites-derived MTs (**Supplementary Figure 6C**). Representative histograms illustrated CD39 and CD73 expression in CD10^−^ versus CD10^+^ and CD90^−^ versus CD90^+^ subpopulations (**Figure 5D**). In tumour-derived MTs, both CD39 and CD73 MFI were significantly higher in CD10^+^ than in CD10^−^ cells (CD39, p = 0.0362; CD73, p = 0.0322) and in CD90^+^ than in CD90^−^ cells (CD39, p = 0.0012; CD73, p = 0.0008). In ascites-derived MTs, CD39 MFI was significantly higher in CD10^+^ than in CD10^−^ cells (p = 0.0099), whereas CD73 did not differ significantly between these populations. Neither CD39 nor CD73 expression differed significantly between CD90^−^ and CD90^+^ cells in ascites-derived MTs (**Figure 5E**).

Overall, expression of selected plasticity-associated markers, including CD10, CD24, and CD90, was associated with higher expression of immunoregulatory molecules such as PD-L1, CD47, CD39, CD73, and calreticulin.

### Autologous co-culture for functional immune testing in SOC

Next, we explored the feasibility of functional immune testing using patient-derived MTs and the autologous immune-cell-containing <40 µm fraction. To assess immune-cell content and checkpoint expression, the <40 µm fraction was analysed by flow cytometry in 14 SOC samples following tissue preparation (see Methods). In 9 of 14 cases, more than 50% of viable cells were CD45^+^ immune cells. Among these samples, sufficient material for additional baseline checkpoint analysis and subsequent autologous co-culture was available in five cases. In these five samples, PD-1 and TIGIT expression was assessed on CD8^+^ T cells before initiation of the co-culture experiments (**Figure 6A**).

**Figure 6:**
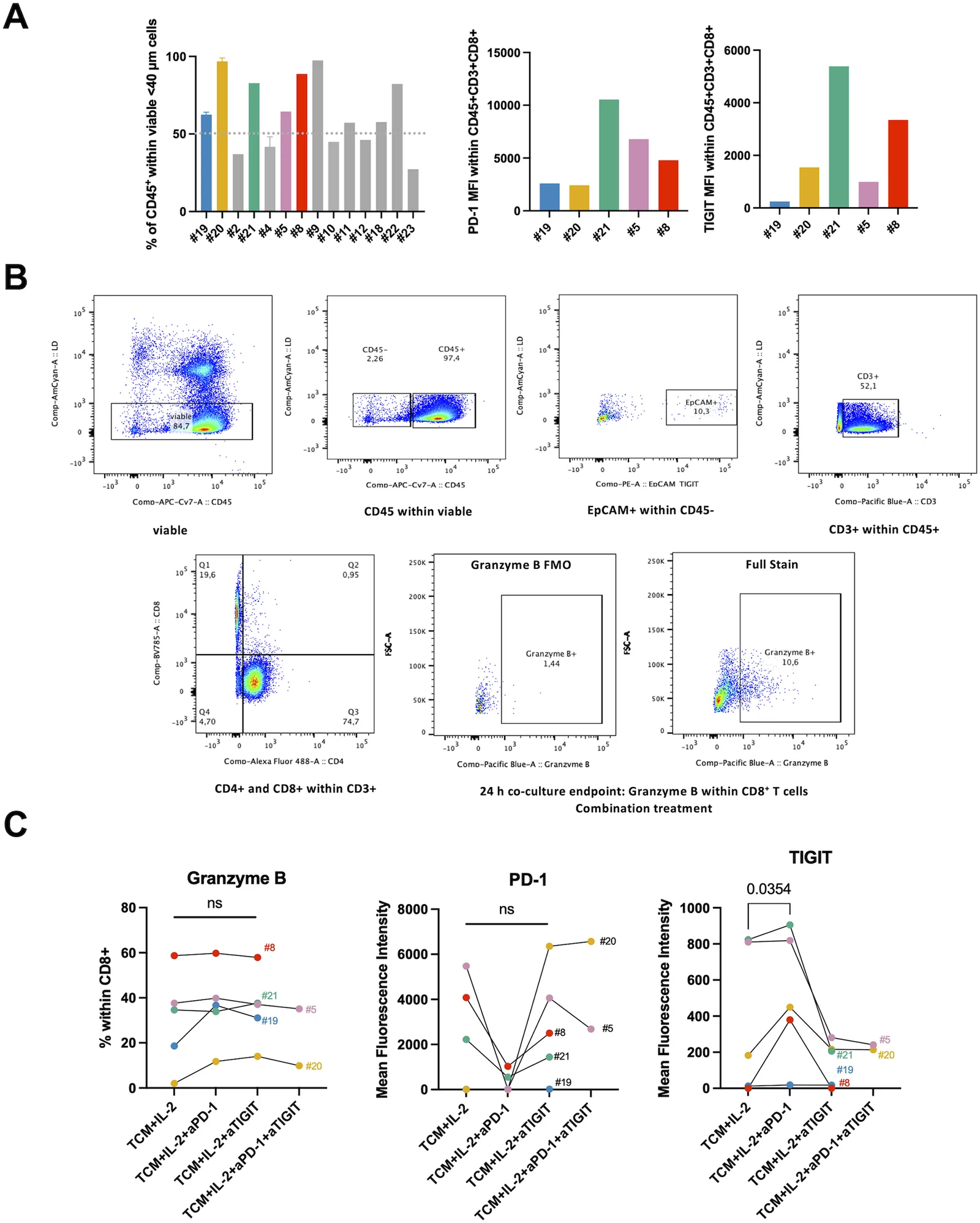
Autologous co-culture of SOC microtumours with matched <40 µm cell fraction. **(A)** Flow cytometric assessment of the proportion of viable CD45^+^ cells within the < 40 µm fraction from 14 SOC patient samples. The dashed line indicates 50% CD45^+^ cells within the viable population. For the five samples included in the autologous co-culture experiments, PD-1 and TIGIT expression on CD45^+^CD3^+^CD8^+^ T cells is additionally shown as mean fluorescence intensity (MFI). These samples are colour-coded consistently across panels **(A, C)**. **(B)** Representative flow-cytometric gating strategy shown for patient #20. The baseline <40 µm fraction was sequentially gated to identify viable cells, CD45^+^ immune cells, EpCAM^+^ cells within the CD45^−^ population, CD3^+^ cells within the CD45^+^ population, and CD4^+^ and CD8^+^ subsets within CD3^+^ T cells. Granzyme B gating within CD8^+^ T cells is shown for the corresponding patient after 24 h autologous co-culture under combination treatment, together with the respective fluorescence-minus-one (FMO) control. **(C)** Flow cytometric analysis of CD8^+^ T cells after 24 h autologous co-culture of MTs and matched <40 µm cell fractions from five patient samples under different treatment conditions (control, anti-PD-1, anti-TIGIT, and combination). Granzyme B expression is shown as the percentage of Granzyme B^+^ cells within the CD8^+^ T-cell population, whereas PD-1 and TIGIT expression are shown as MFI. Each line represents one patient sample, with colours corresponding to those used in panel **(A)** Control, anti-PD-1, and anti-TIGIT conditions were assessed in all five samples, whereas the combination treatment was assessed in two samples and is shown descriptively.

A representative gating strategy for the baseline characterisation of the <40 µm fraction is shown for patient sample #20, illustrating the identification of viable CD45^+^ immune cells, CD3^+^ T cells, CD4^+^ and CD8^+^ T-cell subsets, and EpCAM^+^ cells within the CD45^−^ population (**Figure 6B**). The panel additionally shows representative gating of Granzyme B^+^ cells within CD8^+^ T cells from the same patient after 24 h autologous co-culture, together with the corresponding FMO control.

After 24 hours, flow cytometry showed heterogeneous changes in the frequency of Granzyme B^+^ CD8^+^ T cells across the five autologous co-cultures. In samples #19 and #20, Granzyme B^+^ frequencies increased following both anti-PD-1 and anti-TIGIT treatment compared with the respective control condition. In sample #20, the Granzyme B^+^ frequency also remained above the control level under combined blockade, although the increase was less pronounced than with either monotherapy. In contrast, samples #5, #8, and #21 showed no substantial treatment-associated changes. Consistent with this interpatient heterogeneity, no significant overall difference was detected among the control, anti-PD-1, and anti-TIGIT conditions using the Friedman test (**Figure 6C**).

PD-1 expression varied between samples, with reduced detectable levels following anti-PD-1 treatment and increased levels following anti-TIGIT treatment in a subset of samples, most prominently sample #20; however, the overall difference was not statistically significant. In contrast, TIGIT expression was significantly increased following anti-PD-1 treatment compared with the control condition (p = 0.0354). Reduced detection of the respective target following direct checkpoint blockade may reflect a biological treatment effect and/or impaired flow-cytometric detection due to receptor occupancy or epitope masking. This limitation is particularly relevant for TIGIT, for which the same antibody clone was used for treatment and detection (**Figure 6C**).

## Discussion

The TME in SOC is characterized by profound heterogeneity, immune suppression, and cellular plasticity, all of which contribute to therapy resistance and disease progression. In this study, we used a patient-derived microtumour platform to analyse plasticity-associated markers and immune evasion features in MTs derived from solid tumour tissue and malignant ascites, and to assess the feasibility of autologous MT–immune co-culture as a functional platform for testing immunomodulatory strategies.

In our comparative analysis of tumour- and ascites-derived MTs, expression patterns of established plasticity-associated surface markers (30) were largely conserved across compartments, suggesting a similar stemness-related phenotype within the intraperitoneal tumour environment. CD24 emerged as the most prominently expressed marker, consistent with its association with tumour-initiating potential, metastasis, and chemoresistance in OC (31–33). CD10 and CD133 were also detected in both compartments, in line with reports identifying these markers as indicators of invasive stem-like tumour cell populations (34–36). In OC, CD10^+^/ALDH^+^ cells have been described as apex CSCs capable of generating CD10^−^/ALDH^−^ progeny that form a more chemoresistant compartment (7). The presence of CD10^+^ cells in our treatment-naïve cohort may therefore reflect an early CSC population.

CD44, a transmembrane receptor implicated in metastasis, cancer stem-cell maintenance, and chemoresistance in OC (11), increased significantly in the paired longitudinal analysis and in the unpaired analysis of tumour-derived MTs, whereas the corresponding ascites-derived comparison was not statistically significant. As CD44 is associated with EMT (36), the observed increase may reflect culture-induced modulation towards a more mesenchymal phenotype or the selective persistence of CD44-expressing populations. These possibilities cannot be distinguished using the present relative flow-cytometric data.

At baseline, CD90 expression was descriptively higher and more heterogeneous in solid tumour-derived MTs than in ascites-derived MTs, although this difference was not statistically significant. This observation is consistent with reports describing CD90 enrichment in specific tumour niches influenced by microenvironmental cues (37). In OC, however, CD90 has also been reported to suppress anchorage-independent growth and stem-like properties (12). Previous work from our group demonstrated that ascites-derived ovarian cancer cells retain pronounced tumourigenic capacity and identified mitochondrial oxidative phosphorylation as a potential therapeutic vulnerability in this population (38). However, whether the comparatively low CD90 expression observed in ascites-derived MTs is related to their capacity for anchorage-independent survival or tumourigenicity remains to be determined.

To capture the complexity of plasticity and stemness features, we established a composite plasticity score in which the selected markers were considered together to reflect the overall plasticity phenotype, as individual markers may only partially capture this complex and dynamic cellular state. The score was intended as a descriptive, exploratory measure of combined expression of established plasticity/stemness markers, rather than as a formally validated prognostic or predictive score.

Composite plasticity scoring revealed only modest differences between compartments. Tumour-derived MTs tended to show slightly higher composite plasticity scores than ascites-derived MTs, while the overall marker landscape remained largely conserved, indicating substantial overlap in plasticity-associated phenotypes. Given the cohort-normalized and exploratory nature of the scoring approach, these findings should be interpreted as subtle compartment-associated trends rather than pronounced biological divergence.

Higher composite plasticity scores in tumour-derived MTs may reflect selective pressures within the solid TME, such as hypoxia, matrix stiffness, and immune exclusion, which are known to promote cellular plasticity and aggressive behaviour (39, 40). In contrast, the ascitic microenvironment can promote a hybrid epithelial–mesenchymal phenotype that supports cellular cohesion and phenotypic adaptability, potentially facilitating peritoneal dissemination (41). This may partly explain the slightly lower plasticity scores observed in ascites-derived MTs.

The correlation analyses did not indicate a consistent coordinated expression pattern across the investigated plasticity-associated markers. The strong positive correlation between CD90 and CD133 observed in ascites-derived MTs may point to a subset with combined stemness-associated features. However, because this association was restricted to the small ascites cohort and was not observed in tumour-derived MTs, it should be regarded as exploratory. Representative NxN flow-cytometry plots further illustrated heterogeneous and partially overlapping single-cell marker-expression patterns; however, these representative visualisations cannot establish the prevalence or functional relevance of these patterns across the cohort.

We observed no statistically significant association between higher composite plasticity scores and clinical parameters such as advanced FIGO stage, tumour grade, or residual tumour burden, although trends toward higher scores in more advanced disease were apparent. Given the limited sample size and the predominance of advanced-stage cases in our cohort, these findings should be interpreted as exploratory and hypothesis-generating rather than definitive. Although we did not detect a significant association in our cohort, previous studies have linked increased expression of individual CSC markers, such as CD133, to advanced disease stage and poorer prognosis in OC (42). Furthermore, advanced tumour stage has been associated with lower rates of complete tumour resection (43). Targeting plasticity-associated pathways therefore remains an attractive therapeutic strategy. Inhibitors of EMT signalling, stemness pathways, and microenvironmental factors sustaining plasticity, such as TGF-β or hypoxia-inducible factors, are currently being explored in preclinical models, including OC (44, 45).

At baseline, tumour- and ascites-derived MTs exhibited broadly comparable expression profiles of the investigated immunoregulatory molecules. Among these markers, only calreticulin and CD39 differed significantly at the cohort level, with higher calreticulin and lower CD39 expression in ascites-derived MTs. However, these differences were not reproduced in matched samples, highlighting substantial interpatient heterogeneity rather than a consistent compartment-specific pattern. PD-L1 and PVR showed a trend toward higher expression in ascites-derived MTs, but this did not reach statistical significance, likely reflecting the limited sample size and considerable interpatient variability.

Malignant ascites constitutes a profoundly immunoregulatory environment, characterized by altered cytokine concentrations and enrichment of regulatory T cells and myeloid-derived suppressor cells that can impair antitumor immunity (46–48). The absence of broad differences in tumour-cell-associated marker expression in our study does not exclude these effects. Rather, it suggests that the immunosuppressive properties of ascites may depend substantially on soluble mediators and non-malignant cellular components rather than on a uniformly distinct immunoregulatory phenotype of the epithelial tumour-cell compartment. Although PD-L1 can be induced by inflammatory cytokines within the tumour microenvironment, its expression varied considerably between patients and did not differ significantly between compartments. The lower CD39 expression observed in ascites-derived MTs may indicate a reduced capacity of epithelial tumour cells in ascites to generate immunosuppressive adenosine via the CD39-CD73 axis. However, given that stromal and immune cells also contribute substantially to adenosine production, the overall immunoregulatory impact of this pathway in ascites likely extends beyond tumour-cell-intrinsic CD39 expression (49). Conversely, the higher calreticulin expression in ascites-derived MTs could reflect increased cellular stress or enhanced exposure of a pro-phagocytic “eat-me” signal in the ascites microenvironment. Yet, because CD47 “don´t eat me signal” expression did not differ significantly between compartments and the calreticulin difference was not confirmed in matched samples, the net functional balance between pro-phagocytic and immune-evasive signals remains unclear from these data alone (50).

Downregulation of MHC class I molecules is a well-established mechanism of tumour immune evasion. In serous OC, an inverse correlation between MHC class I expression and PD-L1 levels has been reported, suggesting that tumour cells may employ alternative strategies to avoid immune recognition. Specifically, tumours may either reduce MHC class I expression to impair antigen presentation or upregulate PD-L1 to inhibit cytotoxic T-cell activity (51).

While both solid tumour- and ascites-derived MTs exhibited immune evasive features, these mechanisms were more pronounced in ascites-derived MTs. This suggests that although immune escape is a fundamental characteristic of SOC cells, the ascitic environment may further amplify tumour immunosuppression. Together, these findings highlight the need for therapeutic strategies targeting not only PD-L1 but also alternative immune escape pathways such as CD47 signalling, particularly in patients with advanced ascites-associated disease.

Importantly, our analyses were restricted to viable CD45^−^EpCAM^+^ cells and therefore specifically reflect tumour-cell-associated marker expression. Several of the investigated molecules, including PD-L1, PVR, and CD39, are also expressed by immune and stromal populations and may exert distinct functions within these compartments. In particular, PD-L1 expression on antigen-presenting cells such as dendritic cells can restrain antitumour T-cell responses and contribute to the response to PD-1/PD-L1 blockade (52). These immune-cell-associated contributions were not captured by our tumour-cell-focused analyses and should therefore be considered when interpreting the findings. Previous studies from our group demonstrated compartment-specific expression of TIGIT, PD-1, TIM-3, CD39, and CD73 on ovarian cancer-associated γδ T-cell subsets and identified a TIGIT^+^CD39^+^PD-1^high^ phenotype in conventional CD8^+^ T cells from malignant ascites and tumour tissue (53, 54). The latter study additionally demonstrated CD39 and CD73 expression on EpCAM^+^ ovarian cancer cells, further illustrating the cell-type-dependent distribution of these molecules. However, a parallel lineage-resolved analysis of all markers investigated here was not performed in the present MT cohort. Dedicated analyses will therefore be required to determine how immune and stromal populations contribute to the immunoregulatory properties of SOC MTs. Temporal analysis of CD45^−^EpCAM^+^ MTs from solid tumour tissue and malignant ascites revealed dynamic changes in immune checkpoint marker expression during seven days of *in vitro* culture. While PD-L1 and CD47 levels remained stable, PVR and PVRL2 were significantly upregulated. As absolute MT and tumour-cell numbers were not determined, however, the observed differences between d0 and d7 cannot distinguish genuine modulation of marker expression within tumour cells from selective survival or loss of specific cellular subpopulations. Nevertheless, these findings demonstrate that immune checkpoint expression can change during *in vitro* cultivation, highlighting the importance of assessing the stability of immune checkpoint markers when using such models to investigate therapeutic responses. Changes in checkpoint expression during culture may otherwise influence the apparent response to immune checkpoint-targeted therapies and should therefore be taken into consideration when interpreting results from ex vivo tumour models.

The stability of PD-L1 and CD47 suggests that certain immune evasion mechanisms are maintained during short-term culture, reflecting their constitutive expression in tumour cells. In contrast, increased expression of PVR and PVRL2 may reflect an adaptive response to *in vitro* culture conditions. TIGIT ligands are dynamically regulated in tumour cells and can be induced by stress-associated signalling pathways. For example, activation of the DNA damage response has been linked to increased PVR expression (55).

Similarly, increased calreticulin expression may reflect culture-induced stress or altered cellular homeostasis. Stress-induced calreticulin upregulation has also been reported in lung carcinoma cells following photodynamic therapy (56). The concurrent stability of CD47 suggests a balance between pro- and anti-phagocytic signals that may influence immune cell interactions in co-culture systems.

These observations highlight the importance of considering culture-mediated changes in immune marker expression when designing and interpreting *in vitro* co-culture experiments. During sample preparation, cells <40 µm are typically discarded and MTs are subsequently cultured in media optimized for epithelial tumour cells (see Methods), but not for immune or stromal cell survival. Consequently, immune and stromal cells progressively decline, reducing cytokine and chemokine signals that normally shape tumour–immune interactions. In addition, medium additives such as TGF-β and ROCK inhibitors may influence tumour cell signalling and contribute to the upregulation of PVR, PVRL2, and calreticulin during culture. Together, these factors emphasize the need to carefully consider culture conditions and time points to preserve the translational relevance of co-culture models. Moreover, the distribution and composition of immune cells in peripheral blood and malignant ascites may influence the tumour microenvironment and immune marker expression, representing a potential source of bias not fully captured by the MT system.

Analysis of viable CD45^−^EpCAM^+^ MT cells revealed that the CD10^+^CD24^+^ subpopulation expressed significantly higher levels of PD-L1, CD47, and calreticulin than CD10^−^CD24^−^ cells in both tumour- and ascites-derived samples. These findings identify a tumour-cell subpopulation combining plasticity-associated markers with both inhibitory and pro-phagocytic immune-regulatory signals.

CD24 functions as both a plasticity-associated marker and an immune checkpoint, mediating stem-like behaviour and suppressing phagocytosis (9). Concurrent expression of PD-L1 and CD47 further supports a multifaceted immune evasion strategy by inhibiting T-cell activity and macrophage-mediated phagocytosis. Increased calreticulin expression together with high levels of CD24, CD47, and PD-L1 highlights the interplay between pro- and anti-phagocytic signals within the TME. Coordinated overexpression of CD24, CD47, and PD-L1 has also been reported in hepatocellular carcinoma, supporting a concerted regulatory mechanism that enhances tumour immune resistance (57).

Plasticity-associated subpopulations also differed in their expression of the purinergic markers CD39 and CD73. In tumour-derived MTs, both markers were enriched in CD10^+^ and CD90^+^ cells, whereas in ascites-derived MTs, significant enrichment was restricted to CD39 in the CD10^+^ population. CD39 and CD73 contribute sequentially to extracellular adenosine production, which can suppress T- and NK-cell activity within the TME (24, 58). The observed associations may therefore link selected plasticity-associated phenotypes to components of the adenosinergic pathway, although functional adenosine production was not assessed. Exploratory UMAP analyses provided a descriptive visualisation of heterogeneous and partially overlapping marker distributions; however, these patterns do not establish the presence of distinct cellular subpopulations or their functional relevance.

Together, these findings demonstrate phenotypic heterogeneity among SOC MT cells and identify associations between plasticity-associated and immunoregulatory markers. Functional studies will be required to determine whether these co-expression patterns contribute to immune resistance or represent therapeutically relevant vulnerabilities. If functionally validated, combined targeting of plasticity-associated and immunoregulatory pathways may offer a strategy to address both immune resistance and cancer stem-like tumour-cell phenotypes.

Building on our characterization of tumour-cell immunoregulatory marker expression, we established an autologous co-culture system to evaluate immune responses in OC. This proof-of-concept approach demonstrates the technical feasibility of combining patient-derived MTs with a matched, immune-cell-containing fraction to assess checkpoint-blockade effects *in vitro*. The seven-day pre-culture period was selected to allow parallel culture of the immune-cell-containing <40 µm fraction and the autologous MTs. During this period, the <40 µm fraction was maintained in IL-2-supplemented T-cell medium on anti-CD28-coated plates to support T-cell survival and activation. These conditions were expected to favour a T-cell-enriched population and therefore limit the representation and potential functional contribution of other immune and stromal cell types originally present in the patient TME. Accordingly, the system was designed as an exploratory, T-cell-focused functional co-culture rather than as a faithful reconstruction of the complete tumour microenvironment.

Across the five autologous co-cultures, changes in CD8^+^ T-cell Granzyme B expression following checkpoint blockade were heterogeneous, with no consistent treatment-associated increase observed. This variability in response to anti-PD-1 and/or anti-TIGIT treatment may reflect patient-specific differences in immune-cell composition, activation state, or baseline checkpoint expression. Larger cohorts and more comprehensive and in-depth characterization of the immune-cell compartment will be required to further investigate and substantiate these potential associations. It may also reflect, at least in part, the generally limited and heterogeneous clinical responses to immune-checkpoint inhibition observed in ovarian cancer. Moreover, as checkpoint inhibitors were not part of the standard treatment received by these patients, corresponding clinical response data were unavailable, and the predictive value of the co-culture model could therefore not be assessed. Interestingly, TIGIT expression was significantly increased following PD-1 blockade, whereas the observed increase in PD-1 expression following TIGIT blockade did not reach statistical significance. Detection of the directly targeted receptor following checkpoint blockade may have been affected by receptor occupancy or epitope masking, particularly for TIGIT, for which the same antibody clone was used for treatment and flow-cytometric detection. However, this technical limitation does not account for the observed cross-checkpoint changes, as TIGIT was measured following PD-1 blockade and PD-1 following TIGIT blockade. The TIGIT increase may therefore indicate compensatory regulation between inhibitory pathways and is consistent with the rationale for combined checkpoint inhibition (59, 60).

Taken together, these data provide preliminary evidence that patient-derived MTs can be combined with a matched, pre-cultured immune-cell fraction for functional interrogation. Rather than attempting to fully reconstitute the native immune microenvironment, this exploratory approach establishes a controlled framework for probing selected patient-specific tumour–immune interactions. Patient-derived MTs have previously been shown to predict therapeutically relevant vulnerabilities (61). However, as shown in our study, temporal changes in marker expression during MT maintenance before and/or during co-culture may influence immune responsiveness and should therefore be considered when designing experimental approaches and interpreting the results. Further optimisation, longitudinal profiling of the immune compartment, and systematic validation in larger cohorts will be required to assess reproducibility, interpatient variability, and the potential utility of autologous MT-immune co-cultures for personalised immunotherapy or rational combination strategies in SOC.

Overall, our work highlights the heterogeneity and immune-evasive features of serous ovarian cancer microtumors, revealing spatial and temporal variation in plasticity and immune marker expression during ex vivo culture conditions. Ascites-derived MTs displayed enhanced immunosuppressive characteristics, while short-term *in vitro* culture induced dynamic changes in key immune checkpoints and immunomodulatory molecules. The implementation of autologous MT-immune co-cultures provides preliminary evidence of the technical feasibility of functional immune testing in a patient-specific setting. Together, these findings support patient-derived MTs as an exploratory platform for investigating selected tumour–immune interactions, while further optimisation and validation in larger cohorts are required to assess their potential application in personalised immunotherapeutic strategies in OC.

## Data Availability

The raw data supporting the conclusions of this article will be made available by the authors, without undue reservation.
