# Supplementary material for: Plasticity and immune marker profiling of tumour- and ascites-derived microtumours in serous ovarian cancer: relevance for short-term culture models
Source: Front Immunol. 2026 Oct 7;17:1901291. doi: 10.3389/fimmu.2026.1901291 (PMC13683704; doi:10.3389/fimmu.2026.1901291)
Supplement: Supplementary file 1 [file SupplementaryFile1.pdf]

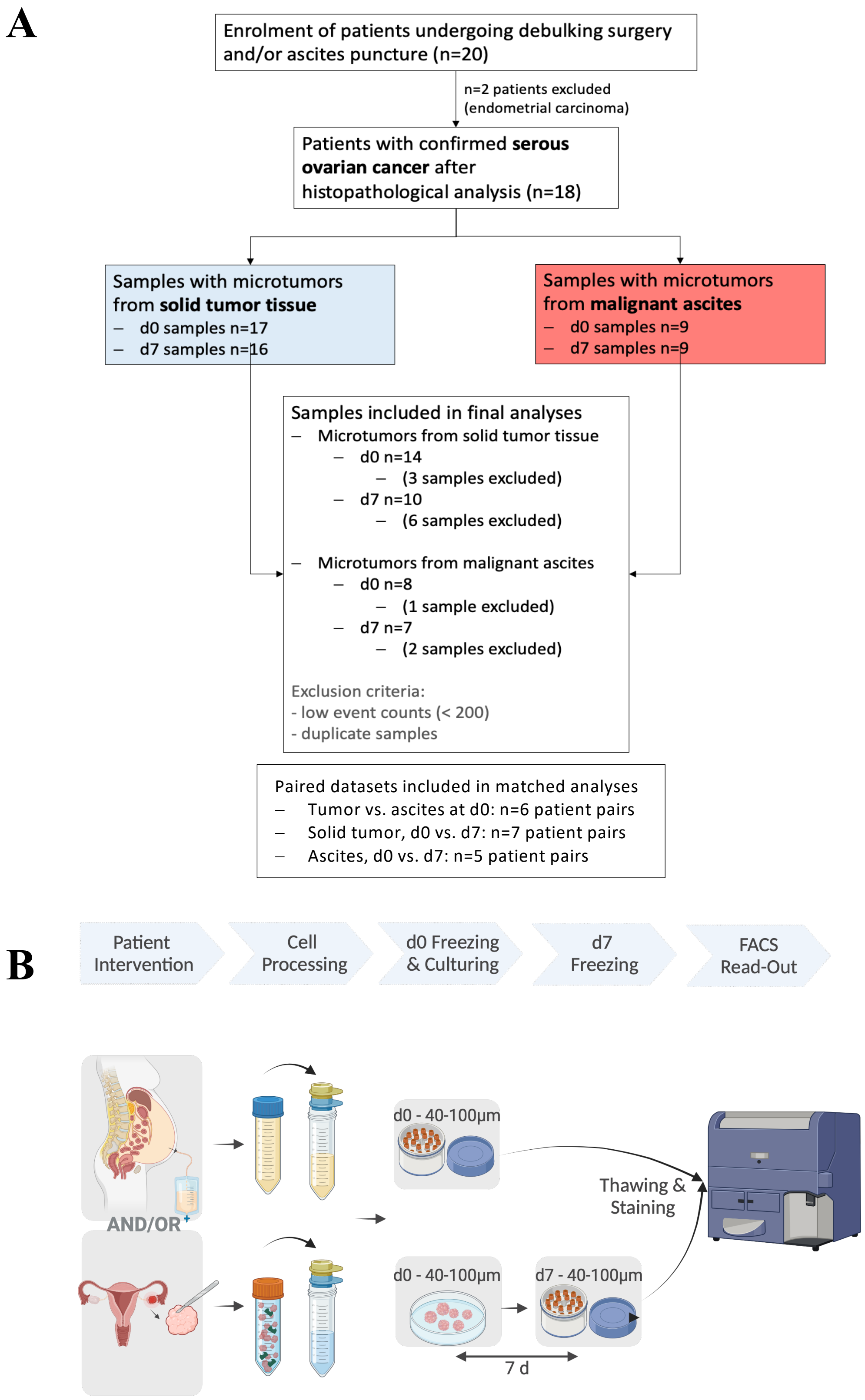

Supplementary Figure 1. Study cohort and sample processing overview.

(A) Cohort selection: From 20 patients undergoing debulking surgery/ascites puncture, 18 with confirmed SOC were analysed after excluding 2 endometrial carcinoma cases. For one patient with duplicate aliquots from the same preparation, only the aliquot with more viable $CD45^{-}EpCAM^{+}$ events was included.

(B) Processing workflow: Tumour tissue (debulking) and ascites (puncture/intraoperative) were mechanically/enzymatically processed, then either cryopreserved immediately (d0: 14 solid/8 ascites) or after 7-day culture (d7: 10 solid/7 ascites). All samples were thawed, stained, and analysed by flow cytometry (40-100µm MTs), excluding samples with <200 events or duplicates. Created with BioRender.com.

Patient number	Diagnosis	Grading	FIGO	Nodal involvement	pT	Residual Tumor	Primary/ recurrent	Plasticity Score	Material	Analysis
#1	HGSOC	hg	IIIC	yes	pT3c	yes	primary	low	T/A	Phenotyping
#2	HGSOC	hg	IV	no	pT3c	yes	primary	high	T/A/<40	Phenotyping+Co-Culture
#3	HGSOC	hg	IVa	no	pT3c	yes	primary	low	T/A	Phenotyping
#4	HGSOC	hg	IIIC	yes	pT3c	no	primary	low	T/A/<40	Phenotyping+Co-Culture
#5	HGSOC	hg	IIIC	yes	pT3c	yes	primary	low	T/A/<40	Phenotyping+Co-Culture
#6	HGSOC	hg	IVa	yes	pT3c	yes	primary	high	T/A	Phenotyping
#7	HGSOC	hg	IIIC	yes	pT3c	no	primary	low	T/A	Phenotyping
#8	SOC	lg	IIIB	no	pT3b	no	primary	high	T/A/<40	Phenotyping+Co-Culture
#9	HGSOC	hg	IIIC	yes	pT3c	yes	primary	*	T/A/<40	Phenotyping+Co-Culture
#10	HGSOC	hg	IIIC	yes	pT3c	no	primary	low	T/A/<40	Phenotyping+Co-Culture
#11	HGSOC	hg	IV	n.k.	pT3c	n.k.	primary	low	T/A/<40	Phenotyping+Co-Culture
#12	HGSOC	hg	IVb	no	pT3c	yes	primary	high	T/A/<40	Phenotyping+Co-Culture
#13	HGSOC	hg	IIIC	n.k.	pT3c	yes	primary	high	T/A	Phenotyping
#14	HGSOC	hg	IIIC	n.k.	n.k.	n.k.	primary	high	T/A	Phenotyping
#15	HGSOC	hg	n.k.	yes	pT3c	yes	primary	high	T/A	Phenotyping
#16	SOC	lg	n.k.	yes	pT3c	no	primary	low	T/A	Phenotyping
#17	HGSOC	hg	IVa	yes	pT3c	yes	primary	*	T/A	Phenotyping
#18	HGSOC	hg	IIIC	n.k.	pT3c	yes	primary	low+high**	T/A/<40	Phenotyping+Co-Culture
#19	HGSOC	hg	IIIC	no	pT3c	yes	primary	n.a.	T/<40	Co-Culture
#20	HGSOC	hg	IIIB	yes	pT3b	no	primary	n.a.	T/<40	Co-Culture
#21	HGSOC	hg	IIIC	yes	pT3c	no	primary	n.a.	T/<40	Co-Culture
#22	HGSOC	hg	IIIC	yes	pT3c	yes	primary	n.a.	T/<40	Co-Culture
#23	HGSOC	hg	IIIC	no	pT3c	yes	primary	n.a.	T/<40	Co-Culture

HGSOC	high-grade serous ovarian cancer
SOC	serous ovarian cancer
hg	high-grade
lg	low-grade
T	tumor-derived microtumors
A	ascites-derived microtumors
<40	<40 µm cell fraction
n.k.	not known
n.a.	not analysed
*	only day 7 samples available; not included in day 7 phenotyping
**	divergent plasticity classification between tumor- and ascites-derived microturmors

Supplementary Table 1. Patient cohorts.

Clinicopathological characteristics of all patients included in the study are shown, including diagnosis, grading, FIGO stage, nodal involvement, pT status, residual tumour status, primary or recurrent disease, plasticity classification, and material used for analysis. In addition to patients included in phenotypic microtumour analyses, samples obtained as part of the autologous co-culture workflow are indicated as "Co-Culture." The <40 µm fraction was characterised in 14 patients. Of these, five patients (#5, #8, and #19–#21) were included in the subsequent functional autologous co-culture experiments, while the remaining samples contributed to baseline characterisation of the immune-cell-containing fraction. Patients #5 and #8 were included in both the phenotypic MT analyses and the co-culture workflow.

A

Antibody	Clone
BV421 CD24	ML5
BV605 CD73	AD2
BV605 HLA-A/B/C	W6/32
BV650 PD-L1	29E.2A3
BV711 EpCAM	9C4
BV785 CD90	5 E10
AF488 CD44	BJ18
PercP-Cy5.5 PVRL2	TX31
PercP-Cy5.5 HLA-/DR/DP/DQ	Tü39
PE CD39	A1
PE Calreticulin	FMC 75
PE/dazzle 594 CD133	clone 7
PE/dazzle 594 PD-L2	MIH18
PE-Cy7 CD10	HI10a
PE-Cy7 PD-1	EH12.2H7
APC PVR	SKII.4
APC CD133	clone 7
AF700 CD47	CC2C6
AF700 CEACAM-1	283340
APC-Cy7 CD45	HI30

B

Antibody	Clone
APC-Cy7 CD45	HI30
BV711 EpCAM	9C4
PE TIGIT	A15153G
PE-Cy7 PD-1	EH12.2H7
FITC/AF488 CD4	SK3
BV785 CD8	RPA-T8
Pacific Blue Granzyme B	GB11
PerCP-Cy5.5 IFNgamma	4S.B3

Supplementary Table 2. Antibody panels and corresponding clones used for phenotypic characterization and autologous co-culture assays.

(A) Flow cytometry panel for characterization of plasticity-associated markers, immune checkpoint molecules, and antigen presentation markers in patient-derived microtumours.

(B) Flow cytometry panel used for functional immune profiling in autologous co-culture assays.

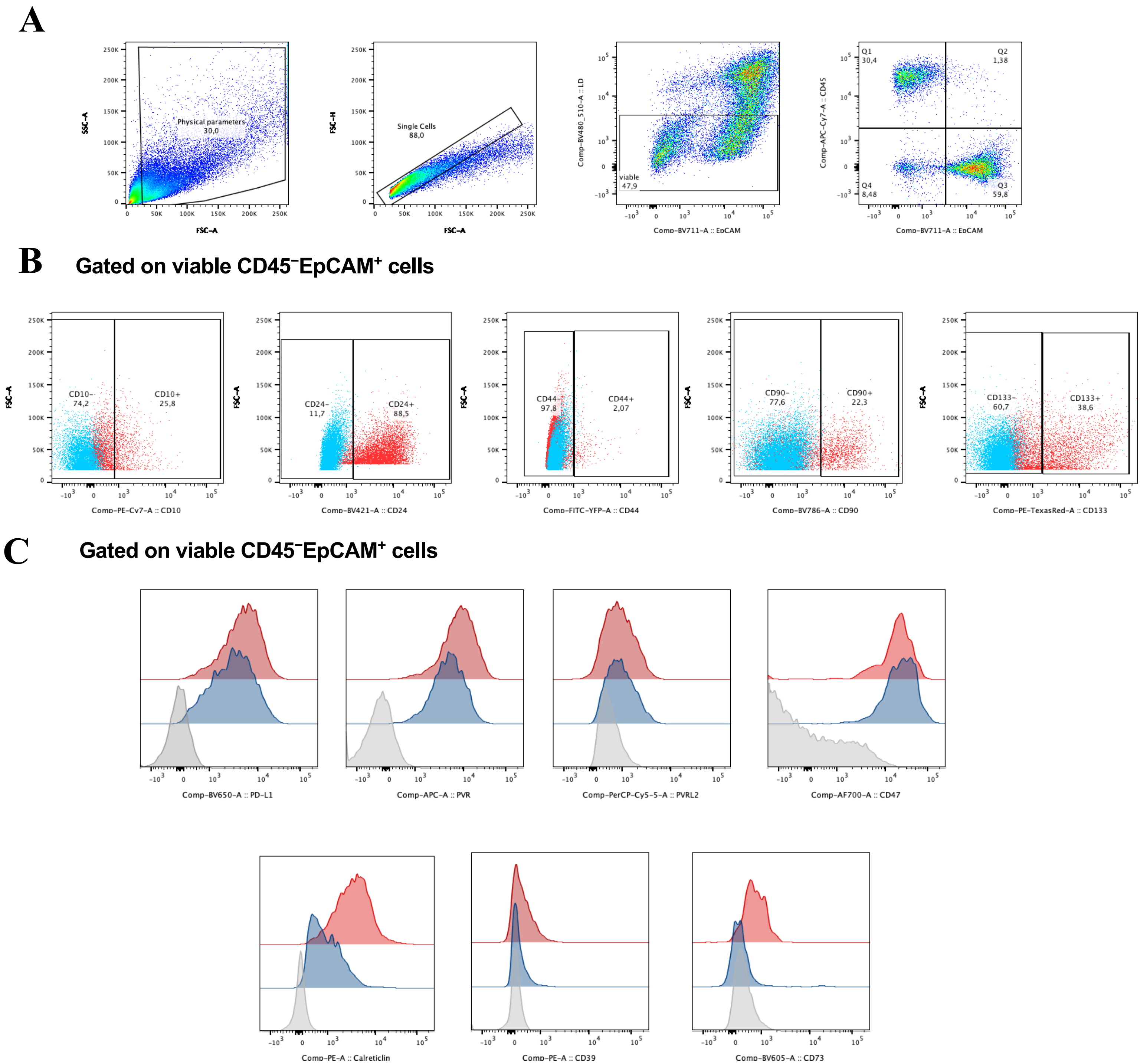

Supplementary Figure 2. Representative gating strategy and flow-cytometric marker-expression profiles of SOC MTs.

(A) Representative sequential gating strategy used to identify viable CD45⁻EpCAM⁺ cells, including morphology-based cell selection, singlet discrimination, exclusion of non-viable cells, and identification of the CD45⁻EpCAM⁺ population. (B) Representative flow-cytometry plots showing CD10, CD24, CD44, CD90, and CD133 expression within viable CD45⁻EpCAM⁺ cells. Events from the fully stained samples are shown in red, and the corresponding fluorescence-minus-one (FMO) controls are overlaid in blue. Marker-positive gates were defined using the respective FMO controls. Numbers indicate the percentages of cells within the displayed gates. (C) Representative normalized fluorescence-intensity histograms showing PD-L1, PVR, PVRL2, CD47, calreticulin, CD39, and CD73 expression in viable CD45⁻EpCAM⁺ tumour-derived (blue) and ascites-derived (red) MT cells, together with the corresponding FMO controls (grey). Histograms were normalized to the modal event count and illustrate the respective marker-expression distributions. Marker expression was quantified as mean fluorescence intensity (MFI) without defining marker-positive gates. Tumour- and ascites-derived samples compared for each marker were acquired within the same flow-cytometry session using identical instrument settings.

A

Patient Number	Tumor(1)/Ascites(2)	% of CD10+	% of CD24+	% of CD90+	% of CD133+	Quartile Scoring
#1	1	25,8	93,2	1,26	1,91	7
#2	1	23,3	88,7	15,1	33,8	11
#3	1	19,7	56	15,2	3,02	7
#4	1	34,6	69	7,14	17,7	9
#5	1	46,8	49,5	68,1	1,88	9
#6	1	30,5	94,3	32,8	3,86	12
#8	1	65,9	67,7	62	37,7	14
#10	1	42,5	65,3	11,7	20,3	10
#11	1	63,9	61,8	39,6	59,2	13
#12	1	28,9	88,9	13,5	28	12
#13	1	65,5	81,7	70	15,6	13
#14	1	24,6	95,6	14,4	27	12
#15	1	31,1	48	77,8	1,03	8
#16	1	51,2	94,8	0,35	4,8	11
#1	2	30,7	88,5	2,59	2,1	8
#4	2	53,4	87,5	14,2	16,6	11
#6	2	63	97,7	7,81	3,02	12
#7	2	28,1	64,8	19,4	38,6	10
#9	2	48,7	88,7	0,15	0,56	8
#10	2	49,9	49,9	12,9	11,9	8
#14	2	27,1	88	14,6	33,9	11
#16	2	22,4	88,4	1,86	6,14	7

	CD10+	CD24+	CD90+	CD133+
**MEDIAN**	32,85	87,75	14,3	13,75
**Q1**	27,35	64,925	7,3075	3,02
**Q2**	32,85	87,75	14,3	13,75
**Q3**	50,875	88,85	29,45	27,75
**Q4**	65,9	97,7	77,8	59,2

	CD10+	CD24+	CD90+	CD133+
**1**	<27,35	<64,925	<7,3075	<3,02
**2**	27,35 – ≤32,85	64,925- ≤87,75	7,3075 – ≤14,3	3,02 – ≤13,75
**3**	32,86 – ≤50,875	87,76 – ≤88,85	14,31 – ≤29,45	13,76– ≤27,75
**4**	>50,88	>88,86	>29,46	>27,76

Quartile Scoring	low (n)	high (n)
d0 tumor	6	8
d0 ascites	5	3

B

Patient Number	Tumor(1)/Ascites(2)	HLA-A/B/C MFI	HLA-DR/DP/DQ MFI
#1	1	957	141
	2	1002	27,4
#2	1	110	10904
#3	1	2046	3147
#4	1	916	294
	2	3995	397
#5	1	4074	8690
#6	1	7187	14174
	2	8770	11542
#7	2	4839	869
#8	1	1765	8628
#9	2	3015	140
#10	1	4066	5981
	2	5404	7102
#11	1	3575	14111
#12	1	1264	1062
#13	1	1555	5476
#14	1	1192	3188
	2	1120	3808
#15	1	1684	1887
#16	1	2704	1613
	2	1642	1014

Median	1905,5	3167,5
M1	<1724,5	<3167,5
M2	>1724,5	>3167,5

HLA-A/B/C-Scoring on d0 CD45-EpCAM+ MT cells

low		high	
Tumor	Ascites	Tumor	Ascites
#1	#1	#3	#4
#2	#15	#5	#6
#4	#18	#6	#7
#13		#8	#10
#14		#11	#11
#15		#12	
#16		#18	
n=7	n=3	n=7	n=5

HLA-DR/DP/DQ-Scoring on d0 CD45-EpCAM+ MT cells

low		high	
Tumor	Ascites	Tumor	Ascites
#1	#1	#2	#6
#3	#4	#5	#11
#4	#7	#6	#15
#13	#10	#8	
#16	#18	#11	
#18		#12	
		#14	
		#15	
n=6	n=5	n=8	n=3

Supplementary Figure 3. Cohort-specific quartile and median thresholds used for plasticity and HLA scoring.

(A) Quartile-based plasticity scoring for d0 $CD45^-EpCAM^+$ MT cells. Expression percentages of CD10, CD24, CD90, and CD133 across all samples were divided into cohort-specific quartiles. Samples were assigned ordinal scores (1–4) according to quartile rank, and composite plasticity scores were calculated by summation of all four markers. Scores of 7–10 were classified as low and 11–14 as high plasticity.

(B) Median-based HLA scoring for d0 $CD45^-EpCAM^+$ MT cells. Cohort-specific median MFI values were calculated for HLA-A/B/C and HLA-DR/DP/DQ. Samples were dichotomized into low (≤ median) and high (> median) expression groups for comparative analyses.

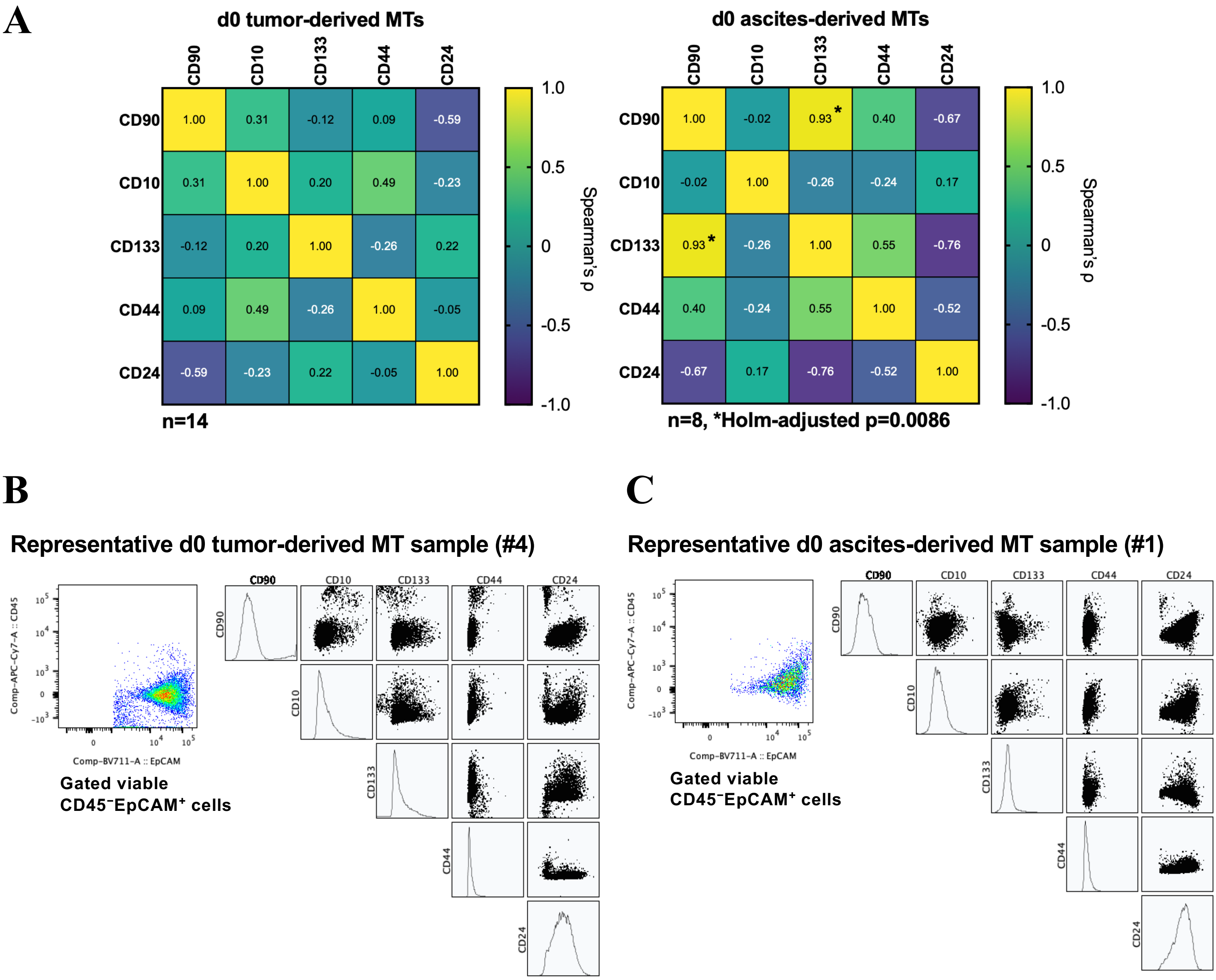

Supplementary Figure 4. Correlation and pairwise expression patterns of plasticity-associated markers in d0 SOC MTs.

(A) Spearman correlation matrices of CD10, CD24, CD44, CD90, and CD133 expression in viable $CD45^{-}EpCAM^{+}$ d0 MT cells derived from solid tumour tissue (n = 14) and malignant ascites (n = 8). Values and colour gradients indicate Spearman's correlation coefficient ρ. Correlations were analysed separately within each compartment using two-sided Spearman correlation tests, and p-values were adjusted across the ten pairwise comparisons using the Holm method. A significant positive correlation between CD90 and CD133 was observed in ascites-derived MTs (p = 0.0086).

(B, C) Representative NxN flow-cytometry plots showing pairwise expression patterns of CD10, CD24, CD44, CD90, and CD133 in gated viable $CD45^{-}EpCAM^{+}$ cells from a tumour-derived MT sample (#4; B) and an ascites-derived MT sample (#1; C). Diagonal plots show the expression distribution of each individual marker, while off-diagonal plots show pairwise marker expression.

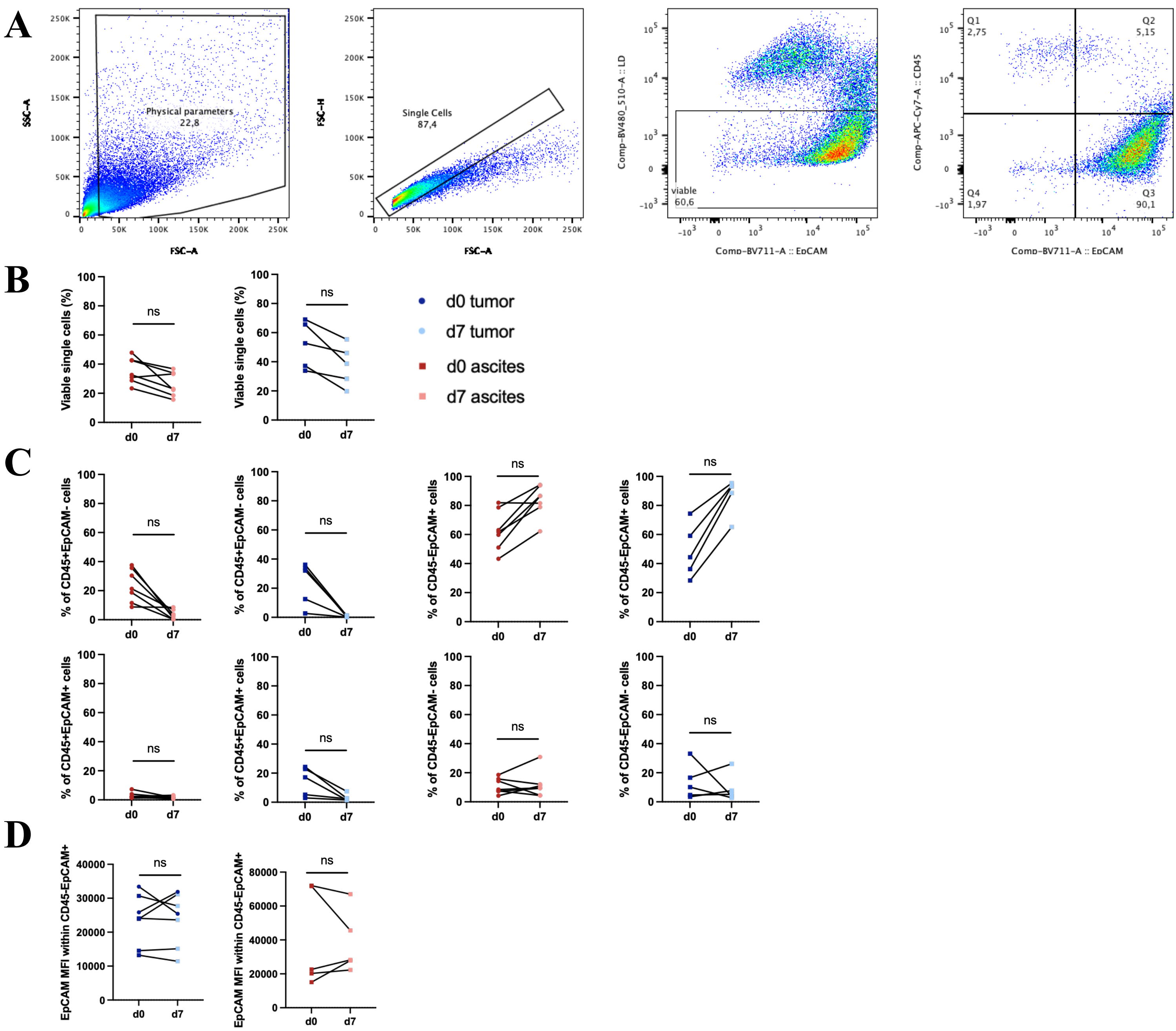

Supplementary Figure 5. Changes in viability and cellular composition of tumour- and ascites-derived SOC MTs during short-term culture.

(A) Representative flow cytometric gating strategy. Cells were sequentially gated based on physical parameters, singlets, and viability, followed by classification of viable single cells according to CD45 and EpCAM co-expression into CD45$^+$EpCAM$^-$, CD45$^-$EpCAM$^+$, CD45$^+$EpCAM$^+$, and CD45$^-$EpCAM$^-$ populations.

(B) Percentage of viable single cells at d0 and d7 in matched ascites-derived (n = 5) and tumour-derived (n = 7) samples.

(C) Proportions of the four CD45/EpCAM-defined populations among viable single cells at d0 and d7. Lines connect matched samples. Dark red and dark blue indicate d0 ascites- and tumour-derived samples, respectively; light red and light blue indicate the corresponding d7 samples. Paired comparisons were performed separately for tumour- and ascites-derived samples using two-sided Wilcoxon matched-pairs signed-rank tests, with p-values adjusted across the five cellular parameters within each compartment using the Holm method. None of the comparisons remained statistically significant after adjustment.

(D) EpCAM mean fluorescence intensity (MFI) within viable CD45$^-$EpCAM$^+$ cells at d0 and d7 in matched tumour- and ascites-derived samples.

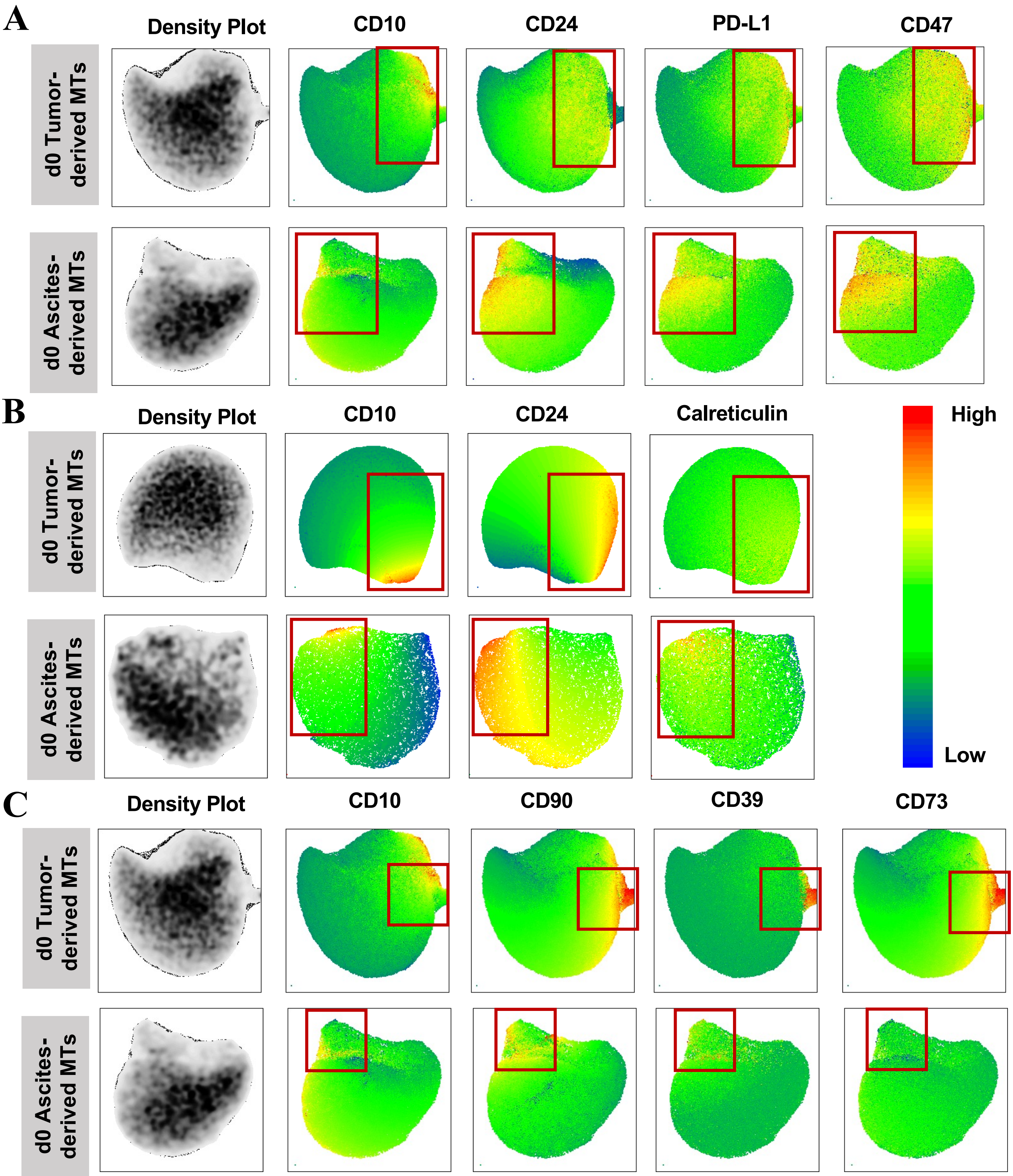

Supplementary Figure 6. Exploratory UMAP visualisation of plasticity-associated and immunoregulatory marker expression in d0 SOC MTs.

UMAPs were generated separately from concatenated viable $CD45^{-}EpCAM^{+}$ cells of solid tumour-derived and ascites-derived d0 MT samples. Density plots indicate the distribution of cells across the respective UMAP embeddings, and colour-coded feature plots display median marker expression from low (blue) to high (red). (A) Spatial expression patterns of CD10, CD24, PD-L1, and CD47. (B) Spatial expression patterns of CD10, CD24, and calreticulin. (C) Spatial expression patterns of CD10, CD90, CD39, and CD73. Panels A and C versus B represent separate flow-cytometry panels and UMAP analyses.
